# Rational Design of Molecularly Imprinted Nanozymes for Selective Degradation of Poly(bisphenol A carbonate) (BPA-PC)

**DOI:** 10.3390/polym18202560

**Published:** 2026-10-17

**Authors:** Tien Tan Bui, Yan Zhao

**Affiliations:** Department of Chemistry, Iowa State University, Ames, IA 50011-3111, USA

**Keywords:** poly(bisphenol A carbonate) (BPA-PC) degradation, enzyme-mimetic catalysis, plastic recycling, molecularly imprinted polymers, polymer hydrolysis, substrate selectivity

## Abstract

Poly(bisphenol A carbonate) (BPA-PC) is an engineering thermoplastic whose recycling is hindered by its Code-7 classification and environmental BPA leaching. Here, we report water-soluble molecularly imprinted nanozymes for the selective depolymerization of BPA-PC under mild alkaline conditions. Synthesized within cross-linked surfactant micelles, the optimized nanozyme combines shape-complementary BPA-matched pockets with surface basicity, achieving a 72-fold rate enhancement over porcine pancreatic lipase. Fluorescence titrations with a soluble dansyl-labeled BPA-PC dimer confirm strong substrate recognition (*K*_a_ ~10^6^ M^−1^). Gel permeation chromatography reveals uniform reductions of 52–55% across M_n_, M_w_, and M_z_, indicative of a random endo-chain cleavage mechanism. On macroscopic BPA-PC films, the nanozyme induces pronounced cracking and surface erosion, whereas polyethylene terephthalate (PET) control films remain undamaged. Furthermore, the aqueous nanozyme retains ~75% of its initial catalytic activity across five consecutive degradation cycles following simple phase extraction. This study demonstrates that tailored, shape-complementary nanozymes provide a generalizable, selective biocatalytic alternative for the circular chemical recycling of recalcitrant engineering plastics.

## 1. Introduction

BPA-PC, an engineering plastic widely used in electronics, auto parts, and consumer goods [1], is produced at >6 Mt per year [2]. Despite its high value, BPA-PC is categorized as a Code-7 “other plastics” under ASTM guidelines, leading to its mixing with chemically diverse polymers during municipal collection. This heterogeneous waste stream renders traditional mechanical recycling economically unviable. Meanwhile, the polymer undergoes slow hydrolytic degradation in landfills and marine environments, leaching monomeric BPA, a known endocrine disruptor [3,4,5].

Selective depolymerization of BPA-PC provides a potential strategy to mitigate BPA pollution by recovering monomers and oligomers from mixed plastic waste for circular reuse. Chemical solvolysis [6,7] can achieve BPA-PC depolymerization at elevated temperatures using organic bases such as N,N′-dimethyl-1,2-diaminoethane [8], 1,5,7-triazabicyclo[4.4.0]-dec-5-ene [9,10], or 1,8-diazabicyclo[5.4.0]undec-7-ene [11]. However, these small-molecule reagents lack the substrate specificity required to selectively target BPA-PC within mixed-plastic streams.

Biocatalysis theoretically resolves this issue with its excellent substrate selectivity [12], yet BPA-PC remains highly recalcitrant to enzymatic [13], fungal [14], or microbial [15] degradation. Unlike structurally flexible aliphatic polycarbonates readily hydrolyzed by lipase [16], BPA-PC has a rigid, highly hydrophobic backbone, sterically restricted by the *gem*-dimethyl quaternary carbon atoms. To date, proficient biocatalysts for BPA-PC degradation remain elusive [17]. Preliminary de novo designed enzymes exhibit minimal activity, constrained to processing soluble, small-molecule model substrates rather than the polymer itself [18].

A critical insight from enzyme engineering is that depolymerization of hydrophobic polymers is strongly governed by enzyme–polymer interfacial interactions [12,19]. To effectively engage a solid, hydrophobic polymer chain, an enzyme catalyst must expose substantial hydrophobic surface area near its active site [20]. Doing so with engineered PETases, for example, requires a delicate balance between installing polymer-recruiting domains and maintaining protein stability. This trade-off poses a severe biophysical challenge for BPA-PC-hydrolyzing enzymes: the rigid, bulky, hydrophobic polymer backbone requires a highly hydrophobic protein surface for effective engagement, yet such surface hydrophobicity also promotes protein aggregation and structural collapse in aqueous media [12].

Molecular imprinting is a method to utilize template molecules and functional monomers (FMs) to form imprinted binding sites inside a cross-linked polymer network [21,22,23,24,25,26,27,28,29,30]. These binding sites can be converted into catalytic active sites upon the installation of catalytic groups [29,31,32,33,34,35]. However, it is impractical to use traditional, insoluble macroporous molecularly imprinted polymers (MIPs) to degrade an insoluble polymer substrate due to challenges in mass transfer. The development of robust, synthetic nanozymes is increasingly recognized as a vital frontier in mitigating plastic pollution, offering stability and reusability that natural enzymes lack under the harsh conditions required to break down rigid consumer plastics [36,37]. Our laboratory has developed a method to prepare water-soluble molecularly imprinted nanoparticles (MINPs) as organic-based nanozymes for different catalytic reactions [38,39,40,41]. We herein report BPA-PC-degrading nanozymes that depolymerize BPA-PC up to 72 times faster than porcine pancreatic lipase, a widely used enzyme for degrading aliphatic polyesters [42], aliphatic polycarbonates [16], and also BPA-PC [13]. Stabilized by covalent cross-links, these molecularly imprinted organic nanozymes remain highly active at elevated temperatures and in mixed organic/aqueous media, conditions that denature most enzyme catalysts. Crucially, the combination of catalyst stability in organic cosolvents with tunable, shape-complementary binding provides a generalizable strategy for the selective chemical recycling of difficult-to-degrade plastics. This approach aims not only to selectively depolymerize recalcitrant plastics within heterogeneous waste streams but also to enable the efficient separation and recovery of degradation products as valuable chemical feedstocks for circular polymer recycling.

## 2. Materials and Methods

### 2.1. General Experimental Methods

All reagents and solvents were of ACS-certified grade or higher and used as received from commercial suppliers. Milli-Q water (18.2 MΩ; Millipore Co., Burlington, MA, USA) was used to prepare buffers and nanozyme catalysts. NMR spectra were recorded on a Bruker DRX-400 (Bruker BioSpin, Billerica, MA, USA), a Bruker AV III 600 (Bruker BioSpin, Billerica, MA, USA), or a Varian VXR-400 spectrometer (Varian, Inc., Palo Alto, CA, USA). High-resolution mass spectra (HR-MS) were recorded on an Agilent QTOF 6540 mass spectrometer with a QTOF detector (Agilent Technologies, Inc., Santa Clara, CA, USA). Dynamic light scattering (DLS) measurements were performed at 25 °C on a Malvern Zetasizer Nano ZS instrument (Malvern Instruments Ltd., Malvern, Worcestershire, UK). TEM imaging was performed on a 200 kV JEOL 2100 scanning/transmission electron microscope (STEM) (JEOL Ltd., Akishima, Tokyo, Japan). UV-vis spectra were recorded on a Cary 50 Bio UV-visible spectrophotometer (Varian, Inc., Mulgrave, Victoria, Australia).

### 2.2. Methods

**Scheme 1:**
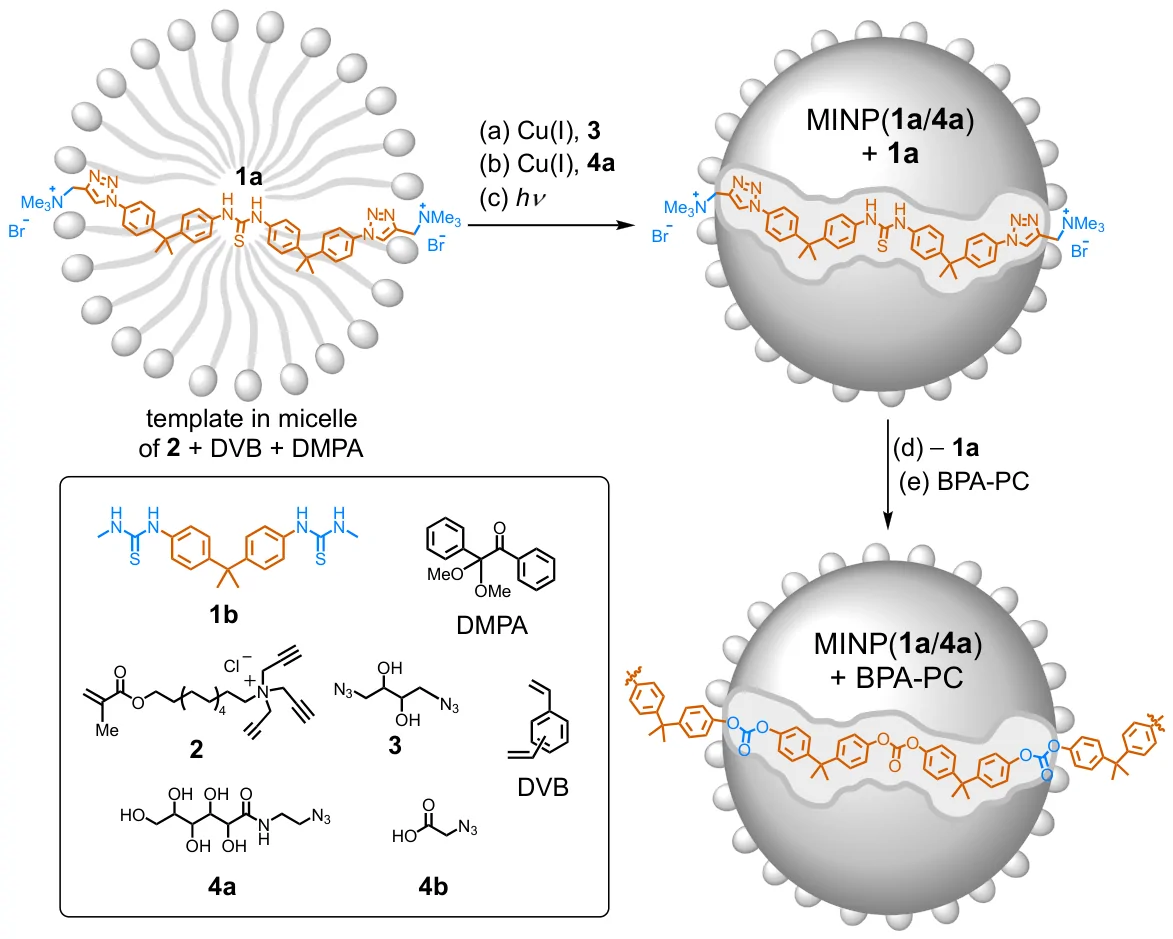
Preparation of MINP(**1a**/**4a**) via templated polymerization of **1a** in the micelle of **2**. The surface ligands are omitted for clarity. Surfactant **2**, diazide surface-cross-linker **3**, and monoazide surface ligand **4a** were synthesized following previously reported procedures [43].

Scheme 1 shows the preparation of BPA-PC-hydrolyzing nanozymes through molecular imprinting within surfactant micelles. Surfactant **2**, diazide surface-cross-linker **3**, and monoazide surface ligand **4a** were synthesized following previously reported procedures [43]. The acylthiourea template molecules **1a** and **1b** contain two and one BPA-mimicking aromatic moieties, respectively, in the middle of the structure (orange-colored). They also possess two (blue-colored) hydrophilic groups on the ends—trimethylammonium for **1a** and *N*-methylthiourea for **1b**. When these amphiphilic molecules are co-solubilized in the micelles of surfactant **2** in water, their hydrophilic groups serve as “anchors” to help the templates reside near the surface of the micelle, due to strong solvation of these groups by water [43]. The expectation is that the resulting imprinted sites will also stay near the surface of the cross-linked micelles, facilitating their interactions with a section of a long polymer chain [39].

The micelles are covered with a layer of terminal alkynes due to the tripropargylammonium head group of **2** and can be cross-linked on the surface by diazide **3** via CuAAC (Scheme 1, step a). Another round of click reaction between monoazide **4a** and the residual alkynes on the micelle surface installs a layer of this hydrophilic ligand on the cross-linked micelles (step b). Molecular imprinting occurs mainly in the third step, when UV irradiation triggers free radical polymerization among the methacrylate groups on the surfactant tails and micelle-solubilized divinylbenzene (DVB) around the templates (step c). The sugar-derived surface ligand **4a** renders the resulting molecularly imprinted nanoparticles (MINPs) insoluble in acetone, allowing the templates to be removed simply by precipitating the aqueous solution with acetone and washing the precipitate with various solvents. MINP(**1a**/**4a**) and MINP(**1b**/**4a**) refer to MINPs prepared using templates **1a** and **1b**, respectively, and surface-functionalized with ligand **4a**. For the MINPs prepared without any surface ligands or with a different ligand (**4b**), the templates are removed by centrifugal filtration and repeated solvent washing. MINPs prepared from **2** have a surfactant aggregation number of ~50 [43]. Thus, a 50:1 surfactant/template ratio in the preparation translates to an average of one binding site per nanoparticle in the final MINP.

#### 2.2.1. Procedure for the Nanozyme Preparation (Scheme 1)

To a micellar solution of compound **2** (8.5 mg, 0.020 mmol) in Milli-Q water (2.0 mL) in a 15 mL glass vial, divinylbenzene (DVB, 1.4 μL, 0.010 mmol), template **1a** or **1b** (10 µL of a 0.040 M solution of corresponding template, 0.00040 mmol), and 2,2-dimethoxy-2-phenylacetophenone (DMPA, 10 μL of a 13 mg/mL solution in DMSO, 0.00050 mmol) were added. The mixture was subjected to ultrasonication for 30 min before compound **3** (3.4 mg, 0.024 mmol), CuCl_2_ (10 μL of a 6.7 mg/mL solution in H_2_O, 0.00050 mmol), and sodium ascorbate (10 μL of a 99 mg/mL solution in H_2_O, 0.0050 mmol) were added.

For the preparation of MINP(**1a**/**4a**), after the reaction mixture was stirred slowly (~60 rpm) at room temperature for 12 h (Scheme 1, step a), compound **4a** (16 mg, 0.060 mmol), CuCl_2_ (10 μL of a 6.7 mg/mL solution in H_2_O, 0.00050 mmol), and sodium ascorbate (10 μL of a 99 mg/mL solution in H_2_O, 0.0050 mmol) were added. After being stirred slowly (~90 rpm) for another 6 h at room temperature (step b), the reaction mixture was sealed with a rubber stopper, purged with nitrogen for 15 min, and subjected to UV irradiation in a Rayonet reactor (~254 nm, 300 W/cm^2^) at room temperature for 16 h (step c). ^1^H NMR spectroscopy was used to monitor the progress of the reaction (Figure S1). The reaction mixture was poured into acetone (8.0 mL). The precipitate was collected by centrifugation and washed with a mixture of acetone/water (5.0 mL/1.0 mL) three times. The crude product was washed additionally with methanol/acetic acid (5.0 mL/0.10 mL) three times and then with excess methanol (2 × 5.0 mL) and acetone (2 × 5.0 mL) (step d). The off-white powder was dried under high vacuum to afford MINP(**1a**/**4a**) (14 mg, 70%). The nonimprinted nanoparticles (NINP) were prepared in a similar manner, except that no template was used.

For the preparation of MINP(**1a**/**4b**), a procedure similar to that described above was followed, except that, after step c, the reaction mixture was subjected to centrifugal filtration through a 10 kDa MWCO filter. The nanoparticles collected were washed with H_2_O:MeOH (1:1, 4 × 5 mL) and MeOH (4 × 5 mL) to afford MINP(**1a**/**4b**).

#### 2.2.2. Preparation of BPA-PC Powder and Film

**BPA-PC powder:** The procedure was adapted from a previous report with slight modifications [15]. BPA-PC pellets (2 g) were dissolved in HPLC-grade dichloromethane (100 mL). Methanol (200 mL) was added dropwise to the stirred solution. The resulting white precipitate was collected by vacuum filtration. The powder obtained was dried under high vacuum at room temperature for 5 h, followed by further drying at 50 °C for 24 h. The resulting powder was used for the degradation studies described in Section 2.2.3.

**BPA-PC film:** The procedure was adapted from a previous report with slight modifications [15]. BPA-PC pellets (0.50 g) were dissolved in HPLC-grade dichloromethane (25 mL). A 5.0 mL aliquot of the solution was cast onto a glass Petri dish (90 mm diameter), and the solvent was allowed to evaporate under ambient conditions to form a thin PC film. The resulting film was carefully removed from the dish using a clean spatula and dried in a fume hood at room temperature for 48 h to ensure the complete removal of residual dichloromethane. The films were subsequently cut into 30 × 30 mm squares and used for the degradation studies detailed in Section 2.2.4.

#### 2.2.3. Characterization of the Degradation of Powdered BPA-PC

**NMR and MS analysis:** A 1-dram vial was charged with BPA-PC powder (5.0 mg) and a magnetic stir bar, followed by the addition of the MINP(**1a**/**4a**) stock solution (25 μL, 500 μM in DMSO), MeOH (37 μL), and CHES buffer (188 μL, 10 mM, pH 10.0). The vial was sealed, placed in a preheated heating block, and stirred at 60 °C for 24 h. After cooling to room temperature, the reaction mixture was diluted with water (450 μL) and CDCl_3_ (700 μL). 1,3,5-Trimethoxybenzene (1.0 μL of a 0.50 M stock solution, 0.50 μmol) was added as an internal standard. The organic layer was separated using a syringe and dried over anhydrous Na_2_SO_4_. A 600 μL aliquot of the dried organic layer was used for ^1^H NMR and positive-ion ESI mass spectrometry (ESI-MS) analysis.

**Gel Permeation Chromatography:** Degradation experiments were performed as described above, except that chloroform (CHCl_3_, 1.0 mL) was used for the extraction. The organic extract was filtered through a 0.22 µm syringe filter prior to analysis. An aliquot of the filtrate was injected onto a TSKgel GMHHR-N column (5 µm, 7.8 mm I.D. × 30 cm, Tosoh Bioscience, Tokyo, Japan) and eluted with CHCl_3_ at 50 °C at a flow rate of 0.5 mL/min. Molecular weight calibration was performed using polystyrene standards (ReadyCal Kit, Sigma-Aldrich, Singapore).

#### 2.2.4. Differential Interference Contrast (DIC) Optical Microscopy

An appropriate plastic film sample (BPA-PC or PET) was placed in a 6-dram vial equipped with a magnetic stir bar, followed by the addition of the appropriate MINP stock solution (40 μL, 500 μM in DMSO), MeOH (210 μL), and CHES buffer (750 μL, 10 mM, pH 10.0). The vial was sealed, placed in a preheated heating block, and stirred at 60 °C for 168 h. After the mixture was cooled to room temperature, the plastic film was removed, transferred to a Petri dish, washed thoroughly with MeOH (5 × 5 mL), blotted dry with a Kimwipe, and further dried in an oven at 60 °C for 48 h. Surface degradation of the film was examined by differential interference contrast (DIC) optical microscopy using a Zeiss Axio Imager Z.2 microscope (Zeiss, Singapore) equipped with ZEN 2 Pro software (blue edition, 2.0.0).

## 3. Results and Discussion

We monitored the formation of MINPs by ^1^H NMR spectroscopy, dynamic light scattering, and transmission electron microscopy (Figures S2–S6). To characterize the imprinted binding sites, we studied the binding of MINP(**1a**/**4a**) or MINP(**1b**/**4a**) to their templates by isothermal titration calorimetry (ITC). Table 1 shows that both nanoparticles bind their corresponding templates strongly, with an apparent binding constant *K*_a_ of 2.1 × 10^5^ M^−1^ for **1a** and 11.9 ×10^5^ M^−1^ for **1b** (entries 1–2). The titration was performed in 5% DMSO in water because the templates have low solubility in water. Even with the organic cosolvent, strong nonspecific interactions were observed with the nonimprinted nanoparticles (NINPs), likely due to the strong hydrophobicity of the BPA-like units in the templates. Despite these complications, MINPs display stronger binding for the templates than NINPs, by 3–7-fold (compare entries 1–2 with 3–4).

We recently reported an MINP-based nanozyme prepared from thiourea **1c** to hydrolyze aryl carbonates [44]. Although substantial rate acceleration has been achieved—i.e., 10^6^ for diphenyl carbonate—the catalyst is unsuitable for BPA-PC degradation. Because the template lacks the “hydrophilic anchors” of **1a** and **1b**, molecular imprinting is expected to create an enclosed hydrophobic imprinted site that cannot bind a long polymer chain. Nonetheless, an important finding in this work is that the polycationic nature of the MINP facilitates the efficient hydrolysis of small-molecule aryl carbonates, due to the surface basicity of the cross-linked micelle [44]. Cationic micelles such as cetyltrimethylammonium bromide (CTAB) are known to facilitate the deprotonation of water by their electric potentials, affording a local pH of 9.5 when the bulk solution is neutral [45]. Cross-linking of the head groups magnifies this effect further [46].


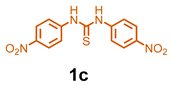


We therefore evaluated MINP(**1a**/**4a**) as a nanozyme for BPA-PC degradation. We hypothesized that the trimethylammonium groups of the template would position two carbonate groups (highlighted in blue in Scheme 1) from a BPA-PC polymer chain close to the micellar surface during the MINP-catalyzed hydrolysis. Surface-associated hydroxide ions, ion-paired with the cationic headgroups of the cross-linked surfactants, could then readily access these nearby carbonate linkages, promoting catalytic hydrolysis of the polymer backbone.

**Figure 1:**
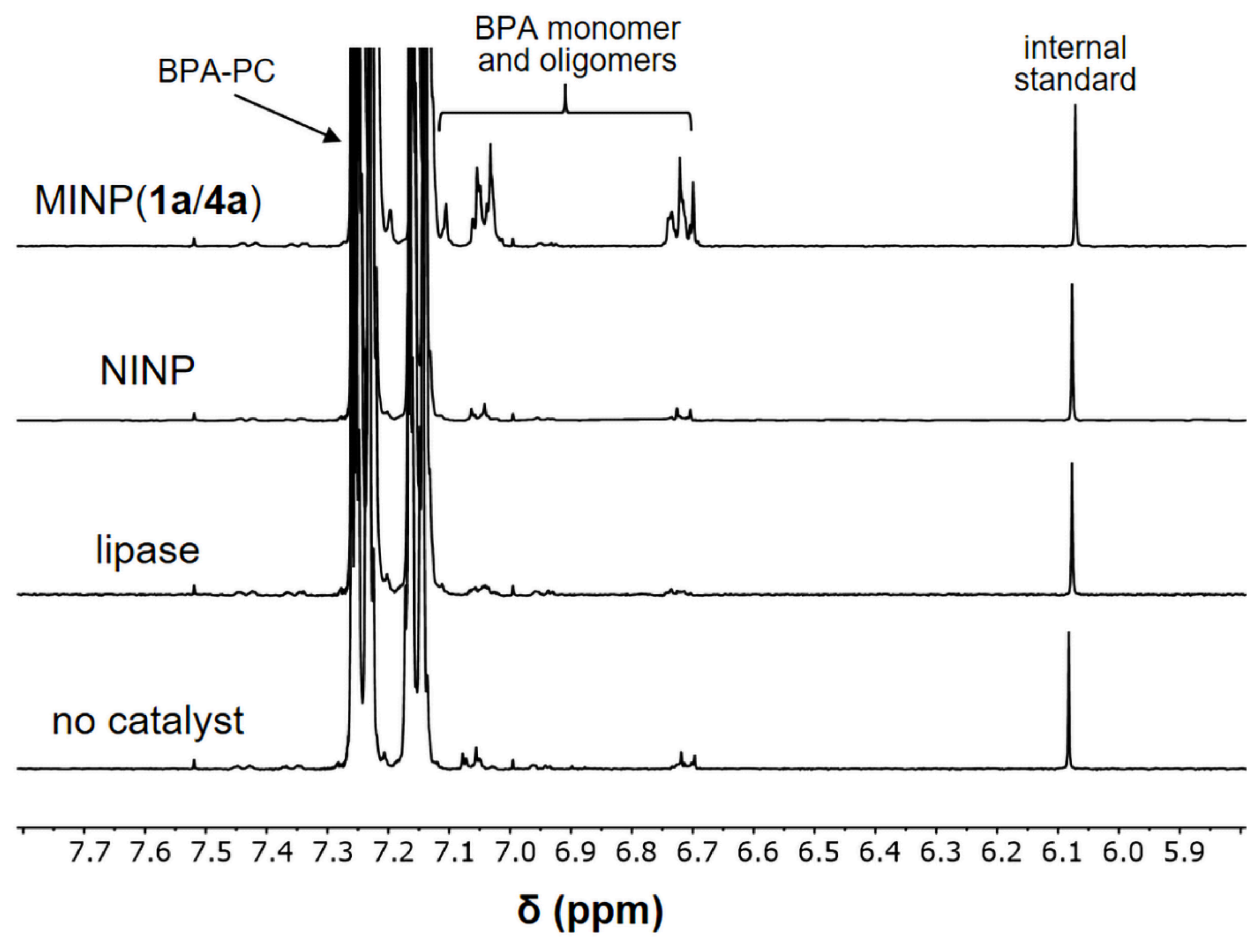
^1^H NMR analysis of polymer degradation by MINP(**1a**/**4a**), NINP, lipase, and without catalyst, after powdered BPA-PC was treated at 60 °C in a 3:1 mixture of pH 10 CHES buffer and organic cosolvents (DMSO + MeOH) for 24 h.

Indeed, when 5.0 mg of powdered BPA-PC was treated with 50 μM MINP(**1a**/**4a**) for 24 h at 60 °C in a 3:1 mixture of pH 10 CHES buffer and organic cosolvents (DMSO + MeOH), a marked increase in BPA monomer and oligomeric products was observed compared to control samples treated with NINP, lipase, or the buffer/methanol mixture alone (Figure 1). Mass spectrometric analysis showed that the oligomeric products consisted predominantly of species ranging from monomer to tetramer (Figure S12). Quantitative analysis by ^1^H NMR spectroscopy and LC–MS revealed that 89.6 ± 0.2% of the BPA-PC remained after 24 h, with monomer and oligomer yields of 0.5 ± 0.1% and 5.6 ± 0.5%, respectively (Table S1, Figures S13 and S14). No methanolysis products were identified during the analysis.

**Figure 2:**
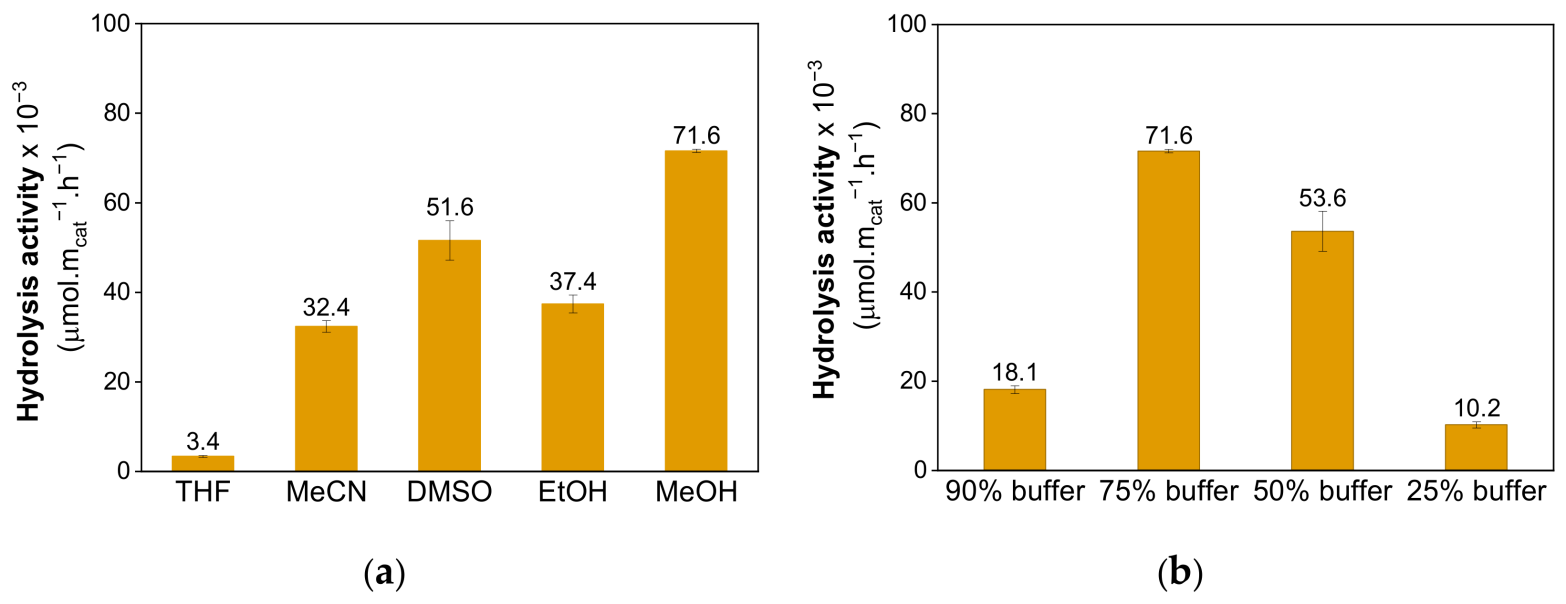
(**a**) Hydrolytic activity of MINP(**1a**/**4a**), measured after powdered BPA-PC was treated at 60 °C for 24 h in a 3:1 mixture of pH 10 CHES buffer and organic cosolvents (DMSO + MeOH). (**b**) Hydrolytic activity of MINP(**1a**/**4a**), measured after powdered BPA-PC was treated at 60 °C for 24 h in different mixtures of pH 10 CHES buffer and methanol. m_cat_ represents the catalyst mass in milligrams.

Organic cosolvents were critical to the degradation reaction. Because the MINP stock solution was prepared in DMSO, every reaction mixture contained ~10 vol % DMSO. On top of that, at a constant 3:1 buffer/organic solvent ratio, the extent of degradation followed the order methanol > DMSO > ethanol ≈ acetonitrile >> THF (Figure 2a). When the amount of buffer in the reaction mixture was varied from 25% to 90%, the highest degradation was observed at 75% buffer, with the rest being DMSO and methanol (Figure 2b). The organic solvents likely swell and soften the BPA-PC chains, thereby increasing chain mobility and improving nanozyme access to carbonate groups at the polymer surface, consistent with previous observations for lipase-catalyzed polyester degradation in organic media [42]. At the same time, binding to the polymer by the MINP is driven largely by hydrophobic interactions [43], which are expected to weaken as the proportion of organic solvents increases. The optimum at a 3:1 buffer/organic solvent ratio therefore most likely reflects a balance between enhanced substrate accessibility and diminished nanozyme–polymer binding.

**Figure 3:**
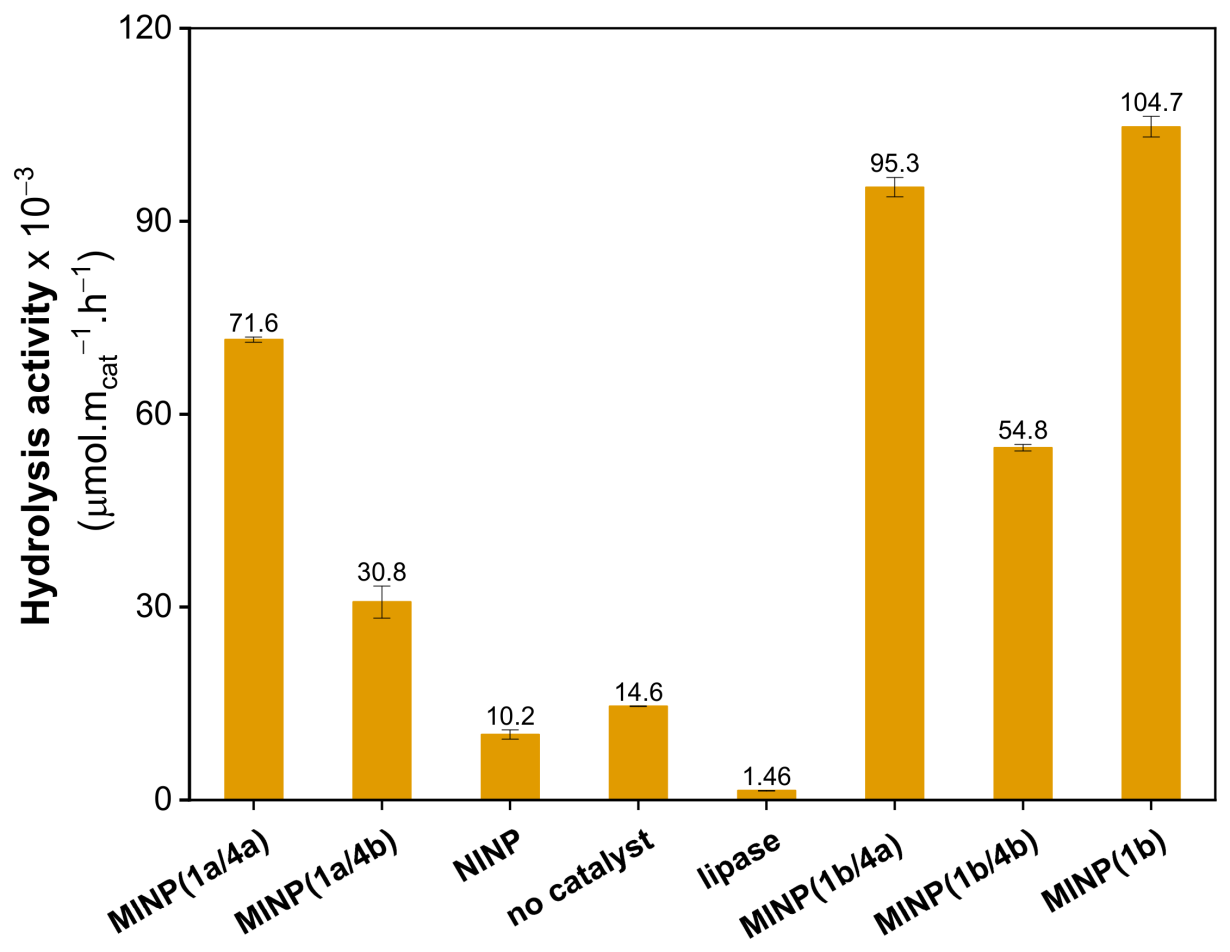
Comparison of BPA-PC degradation activities of different catalysts under the standard catalytic conditions. Polymer degradation was performed in duplicate at 60 °C for 24 h in a 3:1 mixture of pH 10 CHES buffer and organic cosolvents (DMSO + MeOH). m_cat_ represents the catalyst mass in milligrams.

The results so far indicate that the molecularly imprinted binding site on MINP(**1a**/**4a**) is critical to BPA-PC degradation, as NINP exhibited no appreciable catalytic benefit relative to background hydrolysis. Figure 3 shows that MINP(**1b**/**4a**) exhibits 33% higher catalytic activity than MINP(**1a**/**4a**), further supporting the importance of the imprinted site to the catalytic process. Notably, both nanozymes outperform lipase, showing 49- and 65-fold higher activity for MINP(**1a**/**4a**) and MINP(**1b**/**4a**), respectively.

We hypothesized earlier that molecular recognition of the MINP, its surface basicity, and the placement of some carbonate groups of BPA-PC near the surface-associated hydroxide ions collectively are responsible for the polymer degradation. Consistent with the hypothesis, replacing neutral surface ligand **4a** with the carboxylated **4b** reduced the catalytic activities of both MINP(**1a**/**4a**) and MINP(**1b**/**4a**) by 2.3 and 1.7 times, respectively (Figure 3). Once the cationic micelles are functionalized with 2-azidoacetic acid **4b**, the carboxylic acids at pH 10 become anionic carboxylates, neutralizing a substantial amount of positive charge on the micelle surface of MINP(**1a**/**4b**) and MINP(**1b**/**4b**), weakening surface basicity. The reduced electrical potential is evident from the zeta potential of MINP(**1a**/**4a**) vs. MINP(**1a**/**4b**)—35.1 mV and 20.6 mV, respectively. We also tested MINP(**1b**), the nanozyme prepared without any surface ligands. It proved to be the most potent catalyst in this study, exhibiting 72-fold higher degradation activity than lipase. The slightly lower activity of the ligand-functionalized MINP(**1b**/**4a**) suggests that the highly hydrophilic ligands (**4a**) on the nanoparticle surface do not significantly influence polymer–catalyst interactions, likely due to their flexibility.

**Figure 4:**
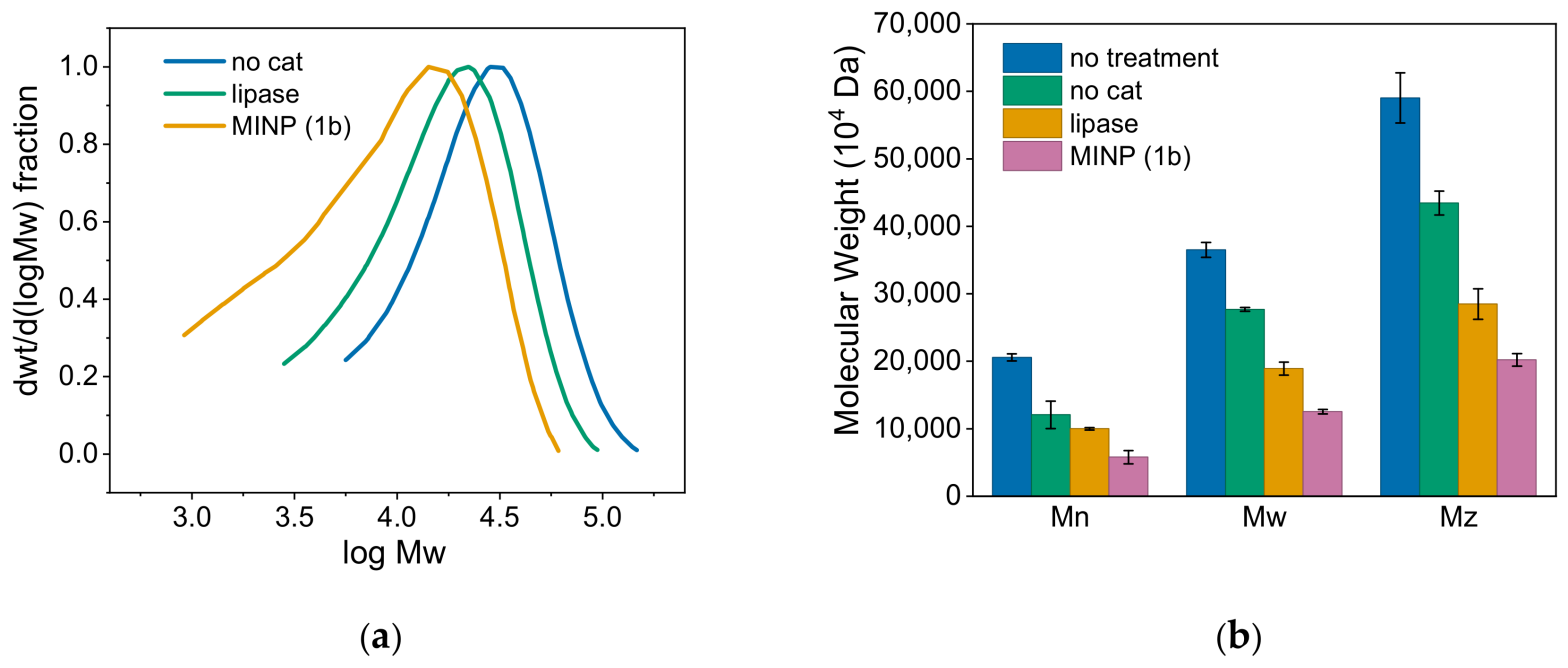
(**a**) Molecular weight distribution of BPA-PC determined by GPC, after the polymer powder was treated at 60 °C in a 3:1 mixture of pH 10 CHES buffer and the organic cosolvents (DMSO + MeOH) for 7 days. (**b**) Molecular weight changes in M_n_, M_w_, and M_z_ of powdered BPA-PC determined by GPC.

Next, gel permeation chromatography (GPC) analysis was used to monitor changes in the molecular weight distribution (MWD) of powdered BPA-PC after 7 days of degradation at 60 °C in a 3:1 mixture of pH 10 CHES buffer and methanol. As shown in Figure 4a, the MWD shifts toward lower molecular weights in the presence of MINP(**1b**), compared to both background and lipase-mediated degradation. In particular, MINP(**1b**) promoted uniform reductions of approximately 52%, 55%, and 54% in M_n_, M_w_, and M_z_, respectively, relative to background degradation (Figure 4b), demonstrating extensive depolymerization of BPA-PC by MINP(**1b**). The proportional decrease across all three molecular weight averages suggests a random, endo-chain cleavage mechanism, where the nanozyme hydrolyzes internal carbonate linkages rather than solely unzipping the polymer from its chain ends [12,47].

**Figure 5:**
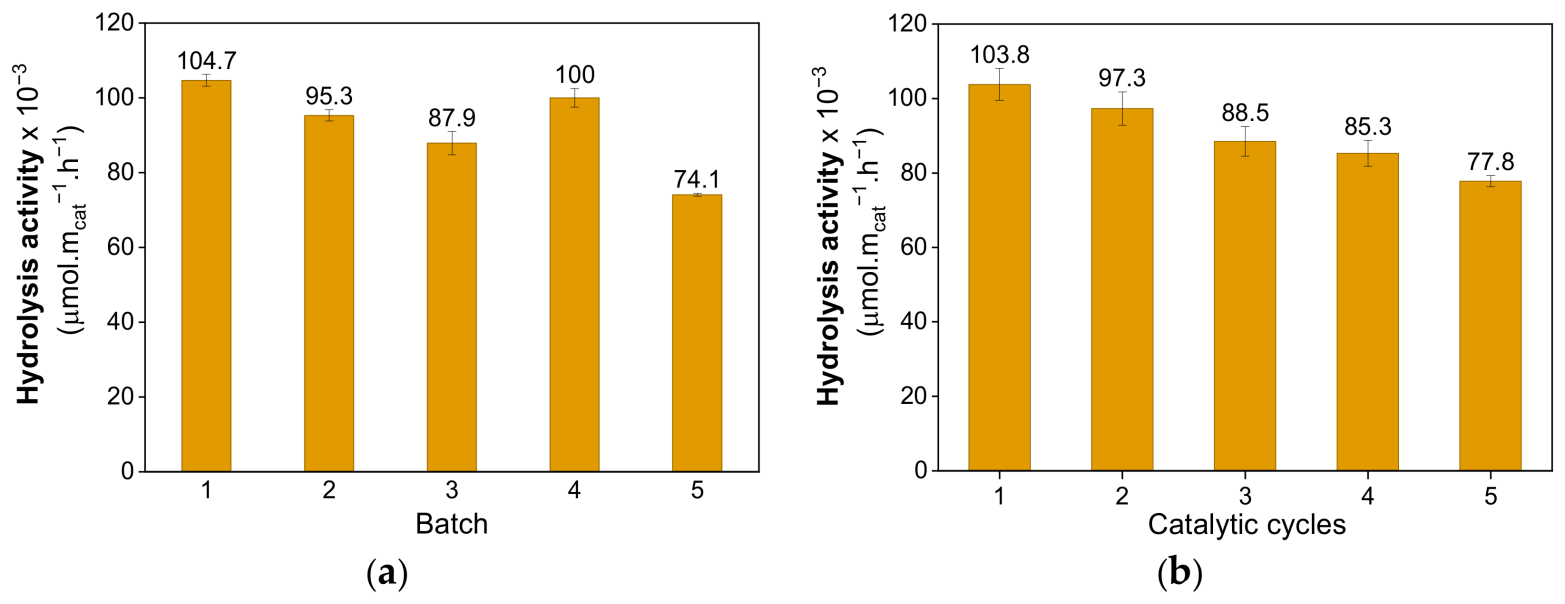
(**a**) BPA-PC degradation activities of different batches of MINP(**1b**) under the standard catalytic conditions. (**b**) Recyclability of MINP(**1b**) in BPA-PC degradation under the standard catalytic conditions. Polymer degradation was performed in duplicate at 60 °C for 24 h in a 3:1 mixture of pH 10 CHES buffer and organic cosolvents (DMSO + MeOH). m_cat_ represents the catalyst mass in milligrams.

Figure 5a shows the activity of BPA-PC degradation for four additional batches (2–5) of MINP(**1b**) in comparison to batch 1 used in Figure 3. The five batches of nanozymes gave an average activity of (92.4 ± 12.0) × 10^−3^ μmol·m_cat_^−1^·h^−1^, indicating good batch-to-batch reproducibility for materials prepared over several months.

To evaluate the potential practical applicability of the catalyst, we performed five consecutive BPA-PC degradations with MINP(**1b**) under our standard conditions. After each cycle, the reaction mixture was extracted with CDCl_3_ (600 μL), and the organic layer was separated to remove remaining BPA-PC and all degraded products from the catalyst in the aqueous phase. The remaining aqueous catalyst phase was heated at 60 °C for 30 min to remove residual organic solvent (CDCl_3_), and its pH was readjusted with 1 M NaOH to pH 10 before addition of a fresh batch of BPA-PC. Despite potential loss of catalyst during this operation and deviation from the initial reaction conditions (e.g., accumulation of salt), the nanozyme retained approximately 75% of its initial degradation activity after five cycles (Figure 5b).

**Figure 6:**
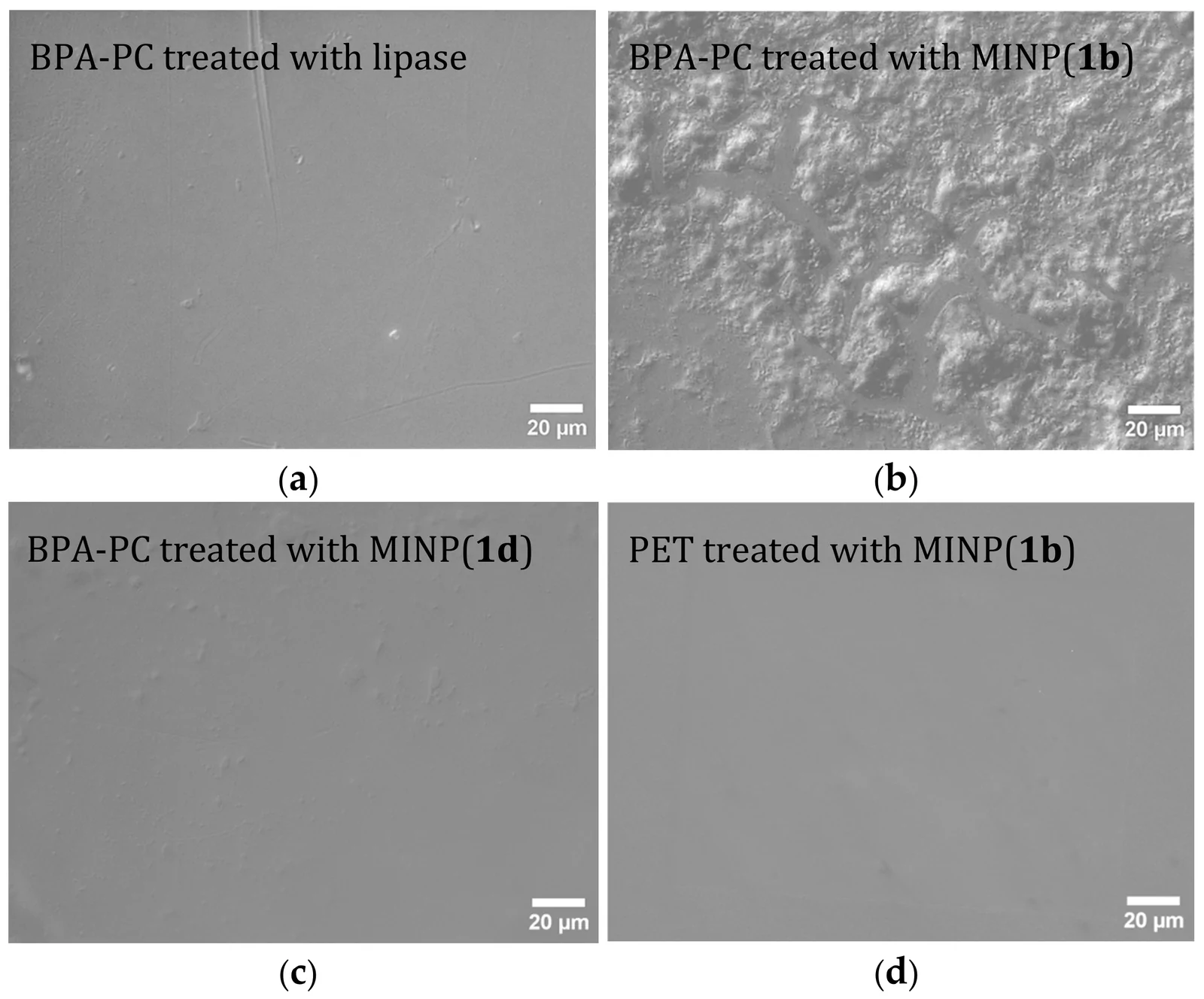
DIC microscopy images of different polymer films after different treatments at 60 °C in a 3:1 mixture of pH 10 CHES buffer and organic cosolvents (DMSO + MeOH) for 7 days. (**a**) BPA-PC film treated with lipase; (**b**) BPA-PC film treated with MINP(**1b**); (**c**) BPA-PC film treated with MINP(**1d**); (**d**) PET film treated with MINP(**1b**).

A longer-term degradation experiment involved treating macroscopic BPA-PC films with MINP(**1b**) for 7 days under our standard reaction conditions. Optical imaging with differential interference contrast (DIC) microscopy shows a smooth, uniform surface without noticeable damage for the lipase-treated control films (Figure 6a). This result aligns with previously reported findings [13]. As discussed earlier, enzymatic degradation of BPA-PC is exceedingly sluggish with most enzymes, and researchers frequently have to resort to high-resolution techniques, such as atomic force microscopy (AFM), to observe minor changes in film morphology following enzymatic treatment [15]. In contrast, the sample exposed to MINP(**1b**) displays pronounced surface roughening and macroscopic cracking under the same conditions (Figure 6b). This morphological damage indicates that the nanozyme successfully promotes localized chain cleavage, resulting in film embrittlement and subsequent stress-induced microscopic fracturing. For our molecularly imprinted nanozymes, specific recognition of the polymer chain by the active site is highly important. To further validate this point, we used template **1d** to create a “skinny” active site for our nanozyme. This bisthiourea molecule is similar to **1b**, except the two geminal methyl groups are replaced with hydrogens. Notably, BPA-PC film treated with the corresponding MINP(**1d**) showed no visible damage after 7 days of treatment (Figure 6c), in sharp contrast to the MINP(**1b**)-treated sample in Figure 6b.


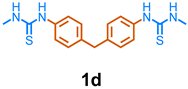


To further confirm the binding-directed degradation, we designed and synthesized a fluorescent “BPA-PC dimer” (**5**), which contains two BPA-like units joined to a dansyl group by a triazole tether. Dansyl is a fluorophore with environmentally sensitive emission [48]. Fluorescence titration indicates strong association between this soluble BPA-PC mimic and MINP(**1a**), with a binding constant of *K*_a_ = 1.08 × 10^6^ M^−1^, corresponding to a binding free energy of −Δ*G* = 8.23 kcal/mol (Table 2, entry 1). MINP(**1b**), our best catalyst, binds with a lower binding constant of *K*_a_ = 0.40 × 10^6^ M^−1^ and a binding free energy of −Δ*G* = 7.64 kcal/mol. The reduced binding for MINP(**1b**) is reasonable, as it can only accommodate one BPA unit, whereas MINP(**1a**) can potentially accommodate two. The important learning here is that stronger binding of the substrate does not necessarily translate into more efficient polymer degradation for these molecularly imprinted nanozymes. Other factors expected to influence polymer degradation include kinetics of substrate binding and product release, accessibility of the polymer chain, and potential product inhibition.


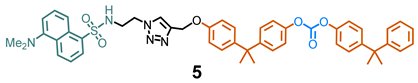


**Table 2:** Binding constants of different nanoparticles for fluorescent BPA-PC dimer **5** determined by fluorescence titration. ^a^

Entry	Host	*K*_a_ (10^6^ M^−1^)	−Δ*G* (kcal/mol)
1	MINP(**1a**)	1.08 ± 0.36	8.23
2	MINP(**1b**)	0.40 ± 0.10	7.64
3	MINP(**1d**)	- ^b^	- ^b^
4	NINP	- ^b^	- ^b^

^a^ The titrations were performed in duplicate in water at 25 °C, and the errors between the runs were <10%. ^b^ The titration data fit poorly to a 1:1 binding model (Figure S7c,d).

Regarding the selectivity among different polymers, MINP(**1b**) produced no visible surface damage on polyethylene terephthalate (PET) films under identical reaction conditions (Figure 6d). This lack of cross-reactivity is another crucial mechanistic indicator. While PET contains hydrolyzable ester bonds, its linear aromatic-aliphatic backbone lacks the bulky *gem*-dimethyl groups and the specific bisphenol repeating units targeted during the imprinting process. Consequently, the PET chains cannot effectively partition into or align within the tailored, shape-complementary hydrophobic pockets of MINP(**1b**). These results suggest that the exceptional substrate selectivity of our catalyst is not merely driven by the nonspecific basicity of the cationic micelle surface, but relies on precise, enzyme-like host-guest recognition at the solid-liquid interface.

## 4. Conclusions

In summary, we report molecularly imprinted nanozymes that combine shape-complementary BPA-like binding pockets with cationic, surface-localized basic sites for selective hydrolysis of BPA-PC under mildly basic conditions. Compared to both background and lipase-catalyzed reactions, the optimized MINP(**1b**) catalyst delivers significantly greater extents of depolymerization, as evidenced by increased release of BPA and oligomers and substantial reductions in M_n_, M_w_, and M_z_ for powdered samples.

The inability of the same nanozyme to degrade PET films highlights its substrate selectivity, which arises from the cooperative action of BPA-matched binding sites and the hydrophobic polymer–micelle interface. This high degree of specificity provides a distinct advantage for real-world applications, as it can be directly exploited to target specific polymers in mixed-plastic waste streams without unwanted cross-reactivity. In the longer term, this strategy supports circular polymer recycling by facilitating the separation of useful chemical feedstocks.

These results demonstrate that molecular imprinting in cross-linkable micelles can emulate key features of polymer-degrading enzymes while overcoming the limitations of natural biocatalysts in terms of stability, operational window, and polymer recognition. Consequently, this method offers a generalizable strategy for upcycling recalcitrant engineering plastics, even those in heterogeneous waste streams.

## Figures and Tables

**Table 1:** Binding data of different nanoparticles for **1a** and **1b** determined by ITC. ^a^

Entry	Host	Guest	*K*_a_ × 10^5^ (M^−1^)	−Δ*G* (kcal/mol)
1	MINP(**1a**/**4a**)	**1a**	2.1 ± 0.1	7.31
2	MINP(**1b**/**4a**)	**1b**	11.9 ± 1.2	8.29
3	NINP	**1a**	0.75 ± 0.02	6.66
4	NINP	**1b**	1.76 ± 0.04	7.16

^a^ The titrations were performed in duplicate using Milli-Q water with 5% DMSO (*v*/*v*) as the solvent. The numbers reported are averages of the two runs. The ITC titration curves are reported in Figures S8–S11.

## Data Availability

The original contributions presented in this study are included in the article/Supplementary Materials. Further inquiries can be directed to the corresponding author.

## Supplementary Materials

The following supporting information can be downloaded at: https://www.mdpi.com/article/10.3390/polym18202560/s1 [49,50,51,52,53,54,55,56,57,58,59,60].

## References

[1] Gilbert E.A., Polo M.L., Maffi J.M., Guastavino J.F., Vaillard S.E., Estenoz D.A. (2022). The organic chemistry behind the recycling of poly(bisphenol-A carbonate) for the preparation of chemical precursors: A review. J. Polym. Sci..

[2] Jehanno C., Alty J.W., Roosen M., De Meester S., Dove A.P., Chen E.Y., Leibfarth F.A., Sardon H. (2022). Critical advances and future opportunities in upcycling commodity polymers. Nature.

[3] Morin N., Arp H.P.H., Hale S.E. (2015). Bisphenol A in Solid Waste Materials, Leachate Water, and Air Particles from Norwegian Waste-Handling Facilities: Presence and Partitioning Behavior. Environ. Sci. Technol..

[4] Wilk B.K., Fudala-Ksiazek S., Szopińska M., Luczkiewicz A. (2019). Landfill leachates and wastewater of maritime origin as possible sources of endocrine disruptors in municipal wastewater. Environ. Sci. Pollut. Res..

[5] La Merrill M.A., Vandenberg L.N., Smith M.T., Goodson W., Browne P., Patisaul H.B., Guyton K.Z., Kortenkamp A., Cogliano V.J., Woodruff T.J. (2020). Consensus on the key characteristics of endocrine-disrupting chemicals as a basis for hazard identification. Nat. Rev. Endocrinol..

[6] Kim J.G. (2020). Chemical recycling of poly(bisphenol A carbonate). Polym. Chem..

[7] Li S., Zhu G., Yang R., Song T., Hou H., Xu G., Wang Q. (2024). Recent advance in poly(bisphenol a carbonate) chemical recycling: Catalysts, reaction strategies, and its applications. Chem. Synth..

[8] Hata S., Goto H., Yamada E., Oku A. (2002). Chemical conversion of poly(carbonate) to 1,3-dimethyl-2-imidazolidinone (DMI) and bisphenol A: A practical approach to the chemical recycling of plastic wastes. Polymer.

[9] Quaranta E., Sgherza D., Tartaro G. (2017). Depolymerization of poly(bisphenol A carbonate) under mild conditions by solvent-free alcoholysis catalyzed by 1,8-diazabicyclo [5.4.0]undec-7-ene as a recyclable organocatalyst: A route to chemical recycling of waste polycarbonate. Green Chem..

[10] Do T., Baral E.R., Kim J.G. (2018). Chemical recycling of poly(bisphenol A carbonate): 1,5,7-Triazabicyclo[4.4.0]-dec-5-ene catalyzed alcoholysis for highly efficient bisphenol A and organic carbonate recovery. Polymer.

[11] Quaranta E., Minischetti C.C., Tartaro G. (2018). Chemical Recycling of Poly(bisphenol A carbonate) by Glycolysis under 1,8-Diazabicyclo[5.4.0]undec-7-ene Catalysis. ACS Omega.

[12] Tournier V., Duquesne S., Guillamot F., Cramail H., Taton D., Marty A., André I. (2023). Enzymes’ Power for Plastics Degradation. Chem. Rev..

[13] Artham T., Mohanalakshmi N., Paragi-Vedanthi P.P., Doble M. (2011). Mechanistic investigations of lipase-catalyzed degradation of polycarbonate in organic solvents. Enzym. Microb. Technol..

[14] Artham T., Doble M. (2010). Biodegradation of Physicochemically Treated Polycarbonate by Fungi. Biomacromolecules.

[15] Yue W., Yin C.-F., Sun L., Zhang J., Xu Y., Zhou N.-Y. (2021). Biodegradation of bisphenol-A polycarbonate plastic by *Pseudoxanthomonas* sp. strain NyZ600. J. Hazard. Mater..

[16] Suyama T., Tokiwa Y. (1997). Enzymatic degradation of an aliphatic polycarbonate, poly(tetramethylene carbonate). Enzym. Microb. Technol..

[17] Thiyagarajan S., Maaskant-Reilink E., Ewing T.A., Julsing M.K., van Haveren J. (2022). Back-to-monomer recycling of polycondensation polymers: Opportunities for chemicals and enzymes. RSC Adv..

[18] Holst L.H., Madsen N.G., Toftgård F.T., Rønne F., Moise I.-M., Petersen E.I., Fojan P. (2023). De novo design of a polycarbonate hydrolase. Protein Eng. Des. Sel..

[19] Groseclose T.M., Nguyen H.B. (2025). Recent advances in enzyme engineering for improved deconstruction of poly(ethylene terephthalate) (PET) plastics. Commun. Mater..

[20] Biundo A., Ribitsch D., Steinkellner G., Gruber K., Guebitz G.M. (2017). Polyester hydrolysis is enhanced by a truncated esterase: Less is more. Biotechnol. J..

[21] Wulff G., Liu J. (2012). Design of biomimetic catalysts by molecular imprinting in synthetic polymers: The role of transition state stabilization. Acc. Chem. Res..

[22] Shen X., Huang C., Shinde S., Jagadeesan K.K., Ekström S., Fritz E., Sellergren B. (2016). Catalytic Formation of Disulfide Bonds in Peptides by Molecularly Imprinted Microgels at Oil/Water Interfaces. ACS Appl. Mater. Interfaces.

[23] Yuan Y., Yang Y., Faheem M., Zou X., Ma X., Wang Z., Meng Q., Wang L., Zhao S., Zhu G. (2018). Molecularly Imprinted Porous Aromatic Frameworks Serving as Porous Artificial Enzymes. Adv. Mater..

[24] Pan J., Chen W., Ma Y., Pan G. (2018). Molecularly imprinted polymers as receptor mimics for selective cell recognition. Chem. Soc. Rev..

[25] Li S., Lieberzeit P.A., Piletsky S., Turner A.P.F. (2019). Smart Polymer Catalysts and Tunable Catalysis.

[26] Zhang H. (2020). Molecularly Imprinted Nanoparticles for Biomedical Applications. Adv. Mater..

[27] Haupt K., Medina Rangel P.X., Bui B.T.S. (2020). Molecularly Imprinted Polymers: Antibody Mimics for Bioimaging and Therapy. Chem. Rev..

[28] Muratsugu S., Shirai S., Tada M. (2020). Recent progress in molecularly imprinted approach for catalysis. Tetrahedron Lett..

[29] Li J., Zhu M., Wang M., Qi W., Su R., He Z. (2020). Molecularly imprinted peptide-based enzyme mimics with enhanced activity and specificity. Soft Matter.

[30] Wei W., Thakur V.K., Chew Y.J., Li S. (2020). Towards next generation “smart” tandem catalysts with sandwiched mussel-inspired layer switch. Mater. Today Chem..

[31] Wei W., Zhou T., Wu S., Shen X., Zhu M., Li S. (2018). An enzyme-like imprinted-polymer reactor with segregated quantum confinements for a tandem catalyst. RSC Adv..

[32] Tang M., Wan J., Wang Y., Yan Z., Ma Y., Sun J., Ding S. (2022). Developing a molecularly imprinted channels catalyst based on template effect for targeted removal of organic micropollutants from wastewaters. Chem. Eng. J..

[33] Tian R.Z., Li Y.J., Xu J.Y., Hou C.X., Luo Q., Liu J.Q. (2022). Recent development in the design of artificial enzymes through molecular imprinting technology. J. Mater. Chem. B.

[34] Piletsky S.A., Piletsky S.S. (2025). Molecularly imprinted polymers as artificial catalysts—Recent trends and emerging applications. TrAC Trends Anal. Chem..

[35] Zhang Z., Luo E., Wang W., Huang D., Liu J., Du Z. (2025). Molecularly imprinted nanozymes with substrate specificity: Current strategies and future direction. Small.

[36] Wan P.P., Chen G.H., Fan J.S., Tan W.L., Li X., Chen L., Li K. (2025). Modulating ion migration realizes both enhanced and long-term-stable nanozyme activity for efficient microplastic degradation. Chem. Sci..

[37] Johnson N.W., Jiang S.Y., Patterson S.B.H., Hinchcliffe T., Vilela F., Yiu H.H.P. (2025). Nanomaterial scaffolds for enzymatic polymer degradation: A tool to advance current biodegradation assessments of polymers in liquid formulation. Biosci. Nanotechnol..

[38] Arifuzzaman M.D., Bose I., Bahrami F., Zhao Y. (2022). Imprinted polymeric nanoparticles as artificial enzymes for ester hydrolysis at room temperature and pH 7. Chem Catal..

[39] Zangiabadi M., Zhao Y. (2022). Synergistic Hydrolysis of Cellulose by a Blend of Cellulase-Mimicking Polymeric Nanoparticle Catalysts. J. Am. Chem. Soc..

[40] Lakavathu M., Zhao Y. (2025). Turning Enzyme Models into Model Enzymes in a Substrate-Tailored, Desolvated Active Site. ACS Catal..

[41] Lakavathu M., Zhao Y. (2025). Cleaving Cellulose into Oligomers with Controllable Chain Lengths Using Synthetic Mimics of Cellulases. ACS Sustain. Chem. Eng..

[42] Kobayashi S., Uyama H., Takamoto T. (2000). Lipase-Catalyzed Degradation of Polyesters in Organic Solvents. A New Methodology of Polymer Recycling Using Enzyme as Catalyst. Biomacromolecules.

[43] Awino J.K., Zhao Y. (2013). Protein-Mimetic, Molecularly Imprinted Nanoparticles for Selective Binding of Bile Salt Derivatives in Water. J. Am. Chem. Soc..

[44] Bui T.T., Zhao Y. (2025). Molecularly Imprinted Nanozymes for Selective Hydrolysis of Aromatic Carbonates Under Mild Conditions. Nanomaterials.

[45] Roy D., Karmakar R., Mondal S.K., Sahu K., Bhattacharyya K. (2004). Excited state proton transfer from pyranine to acetate in a CTAB micelle. Chem. Phys. Lett..

[46] Chadha G., Zhao Y. (2013). Properties of surface-cross-linked micelles probed by fluorescence spectroscopy and their catalysis of phosphate ester hydrolysis. J. Colloid Interface Sci..

[47] Bocharova V., Drufva E.E., Cahill J.F., Popov I., Dishner I.T., Zhou M.C., Jung G.S., Ullman A.M., Carper D.L., Damron J.T. (2025). Factors modulating the hydrolysis of Nylon-6,6 by a nylon hydrolase enzyme. Polym. Chem..

[48] Zhong Z., Zhao Y. (2008). Controlling the conformation of oligocholate foldamers by surfactant micelles. J. Org. Chem..

[49] Seuring J., Reiss P., Koert U., Agarwal S. (2010). Synthesis, characterization and properties of a new polymerisable surfactant: 12-Methacryloyl dodecylphosphocholine. Chem. Phys. Lipids.

[50] Zhao Y., Zhong Z. (2005). Oligomeric Cholates: Amphiphilic foldamers with nanometer-sized hydrophilic cavities. J. Am. Chem. Soc..

[51] Xiao Z., Song S., Chen D., van Merkerk R., van Der Wouden P.E., Cool R.H., Quax W.J., Poelarends G.J., Melgert B.N., Dekker F.J. (2021). Proteolysis targeting chimera (PROTAC) for macrophage migration inhibitory factor (MIF) has anti-proliferative activity in lung cancer cells. Angew. Chem. Int. Ed..

[52] Ryu E.H., Zhao Y. (2005). Efficient synthesis of water-soluble calixarenes using click chemistry. Org. Lett..

[53] Lewandowski B., De Bo G., Ward J.W., Papmeyer M., Kuschel S., Aldegunde M.J., Gramlich P.M., Heckmann D., Goldup S.M., D’Souza D.M. (2013). Sequence-specific peptide synthesis by an artificial small-molecule machine. Science.

[54] Schaufelberger F., Seigel K., Ramström O. (2020). Hydrogen-bond catalysis of imine exchange in dynamic covalent systems. Chem.–Eur. J..

[55] Stacy D.M., Le Quement S.T., Hansen C.L., Clausen J.W., Tolker-Nielsen T., Brummond J.W., Givskov M., Nielsen T.E., Blackwell H.E. (2013). Synthesis and biological evaluation of triazole-containing *N*-acyl homoserine lactones as quorum sensing modulators. Org. Biomol. Chem..

[56] Ye Y.S., Huang Y.J., Chang F.C., Xue Z.G., Xie X.L. (2014). Synthesis and characterization of thermally cured polytriazole polymers incorporating main or side chain benzoxazine crosslinking moieties. Poly. Chem..

[57] Silver R., Nirpal A.K., Sathyamoorthi S. (2024). Taming tethered nitreniums for alkene functionalization reactions. J. Org. Chem..

[58] Saura A.V., Marin M.J., Burguete M.I., Russell D.A., Galindo F., Luis S.V. (2015). The synthesis of new fluorescent bichromophoric compounds as ratiometric pH probes for intracellular measurements. Org. Biomol. Chem..

[59] Bahrami F., Zhao Y. (2024). Rational design and synthesis of an artificial enzyme for S_N_2 reactions through micellar imprinting. Org. Lett..

[60] Batey R.A., Santhakumar V., Yoshina-Ishii C., Taylor S.D. (1998). An efficient new protocol for the formation of unsymmetrical tri- and tetrasubstituted ureas. Tetrahedron Lett..

