# Supplementary material for: Rational Design of Molecularly Imprinted Nanozymes for Selective Degradation of Poly(bisphenol A carbonate) (BPA-PC)
Source: Polymers (Basel). 2026 Oct 17;18(20):2560. doi: 10.3390/polym18202560 (PMC13709287; doi:10.3390/polym18202560)
Supplement: Supplementary file 1 [file polymers-18-02560-s001.zip › polymers-4515842-supplementary.pdf]

Supporting Information

Rational Design of Molecularly Imprinted Nanozymes for Selective Degradation of Poly(bisphenol A) Carbonate (BPA-PC)

Tien Tan Bui and Yan Zhao*

Department of Chemistry, Iowa State University, Ames, Iowa 50011-3111, USA

**zhaoy@iastate.edu*

Table of Contents

Reagents and Stock Solutions *S3*
General Experimental Methods *S4*
Synthesis and Characterizations *S4*
Scheme S1. .... *S4*
Scheme S2. .... *S5*
Scheme S3. .... *S5*
Scheme S4. .... *S5*
Characterization of MINPs *S10*
Figure S1. .... *S10*
Dynamic Light Scattering *S11*
Figure S2. .... *S11*
Figure S3. .... *S12*
Figure S4. .... *S12*
Figure S5. .... *S13*
Zeta potential *S14*
Figure S6. .... *S15*
Determination of binding constant for compound 5 using fluorescence titration with different nanoparticles *S15*
Figure S7. .... *S16*
Isothermal Calorimetry (ITC) experiments *S16*
Figure S8. .... *S17*
Figure S9. .... *S17*
Figure S10. .... *S18*

Figure S11. .... S18
Study of degradation of bisphenol A polycarbonate (BPA-PC) S19
Mass spectrum analysis S19
Figure S12. .... S19
Quantitative analysis S19
Table S1. .... S19
Figure S13. .... S20
Figure S14. .... S20
Gel permeation chromatography analysis S21
Figure S15. .... S21
^{1}H and ^{13}C{^{1}H} NMR spectra of key compounds S22
Figure S16. .... S22
Figure S17. .... S22
Figure S18. .... S23
Figure S19. .... S23
Figure S20. .... S24
Figure S21. .... S24
Figure S22. .... S25
Figure S23. .... S25
Figure S24. .... S26
Figure S25. .... S26
Figure S26. .... S27
Figure S27. .... S27
Figure S28. .... S28
Figure S29. .... S28
Figure S30. .... S29
Figure S31. .... S29
Mass spectra S30
Figure S32. .... S30
Figure S33. .... S30
Figure S34. .... S30
Figure S35. .... S31
References S31

Reagents and Stock Solutions

Poly(Bisphenol A)carbonate (BPA-PC)
Polyethylene Terephthalate (PET)
Triphosgene
Pyridine
N, *N'*-Diisopropylethylamine (DIPEA)
Methyl amine
tert-butyl nitrite
Tetrahydrofuran (THF)
Ethyl acetate (EtOAc)
Acetonitrile (MeCN)
Dichloromethane (DCM)
Hexanes
Methanol (MeOH)
Acetone
Acetic acid
1,4-dioxane
Diethyl ether (Et_2O)
Sodium sulfate (anhydrous) (Na_2SO_4)
Sodium carbonate (Na_2CO_3)
Sodium bicarbonate ($NaHCO_3$)
12-(methacryloyloxy)-*N*, *N*, *N*-tri(prop-2-yn-1-yl)dodecan-1-aminium chloride (compound **2**, cationic surfactant containing alkynes)
1,4-diazidobutane-2,3-diol (compound **3**, surface cross-linking azide)
N-(2-azidoethyl)-2,3,4,5,6-pentahydroxyhexanamide(compound **4a**, surface-decorating azide)
Divinylbenzene (DVB)
2,2-dimethoxy-2-phenylacetophenone (DMPA)
Copper (II) chloride ($CuCl_2$)
Copper (I) iodide (CuI)
Sodium ascorbate
Sodium azide (NaN_3)
Nonimprinted nanoparticles (NINPs)
N-cyclohexyl-2-aminoethanesulfonic acid (CHES, for buffer preparation with pH 10.0)

General Experimental Methods

All reagents and solvents were of ACS-certified grade or higher and used as received from commercial suppliers. Millipore water (18.2 MU; Millipore Co., USA) was used to prepare buffers and nanoparticles. All new compounds were characterized by ^{1}H NMR, ^{13}C NMR and HRMS. NMR spectra were recorded on a Bruker DRX-400, a Bruker AV III 600, or a Varian VXR-400 spectrometer. Chemical shifts are reported in ppm relative to residual solvent peaks. Coupling constants are reported in hertz. High-resolution mass spectra (HR-MS) were recorded on Agilent QTOF 6540 mass spectrometer with a QTOF detector. Dynamic light scattering (DLS) was recorded at 25 °C on a Malvern Zetasizer Nano ZS instrument. TEM was analyzed on a 200kV JEOL 2100 scanning/transmission electron microscope (STEM). Gel permeation chromatography was recorded on Agilent PL-GPC 50 with a refractive index detector. High performance liquid chromatography (HPLC) was recorded

Synthesis and Characterizations

BPA-PC was purchased from Sigma–Aldrich (product ID 435139). PET film was purchased from MSE Supplies (thickness 0.035 mm, SKU# GL0450). Syntheses of compounds **2–4a**[1-3], compound **4b**[4], compound **S3**[5] , compound **S5**[9], and compound **S7**[10] followed previously reported procedures.

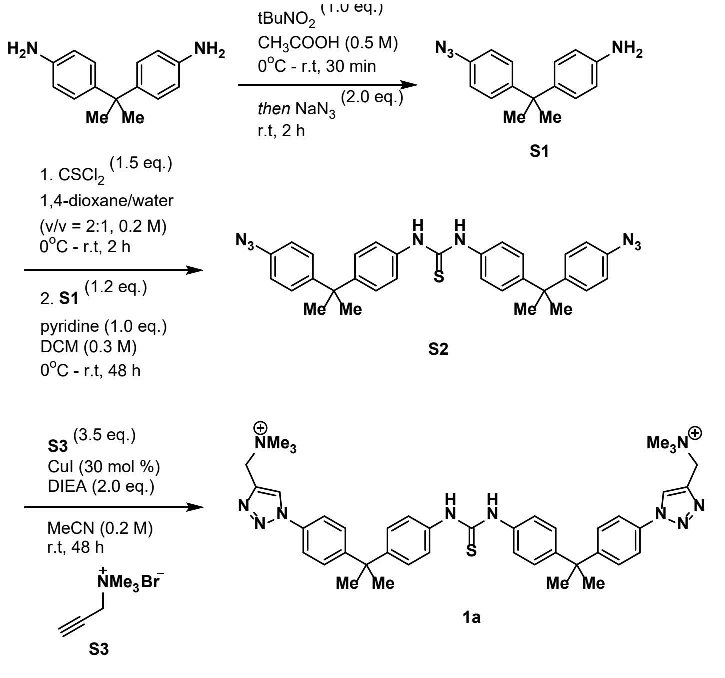

Scheme S1. Synthesis of compound **1a**

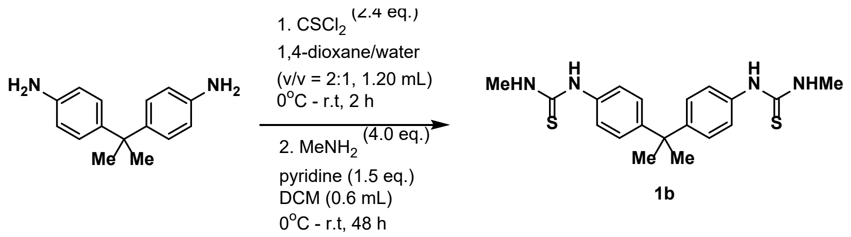

Scheme S2. Synthesis of compound **1b**

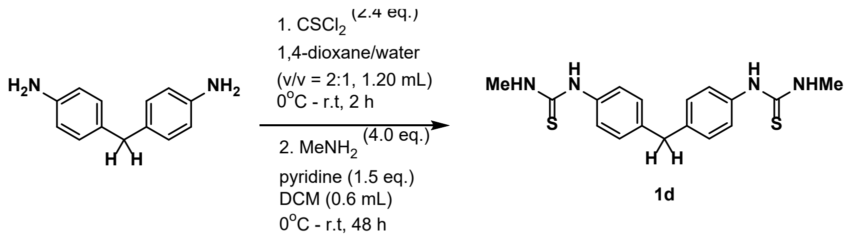

Scheme S3. Synthesis of compound **1d**

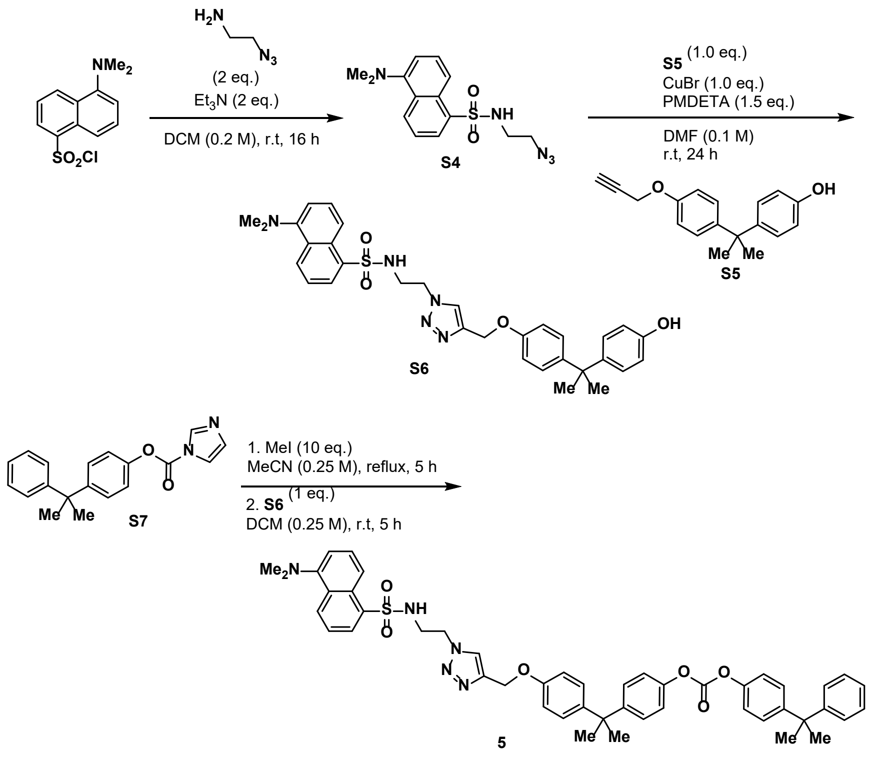

Scheme S4. Synthesis of compound **5**

Compound S1. Compound **S1** was synthesized following a modified literature procedure.[6] 4,4'-(Propane-2,2-diyl)dianiline (1.50 g, 6.5 mmol, 1.0 equiv.) was dissolved in glacial acetic acid (13 mL, 0.50 M) in a 100 mL round-bottom flask and cooled to 0 °C. *tert*-Butyl nitrite (0.86 mL, 6.5 mmol, 1.0 equiv.) was added dropwise to the reaction mixture. The reaction mixture was warmed

to room temperature and stirred for 30 min. NaN_3 (0.90 g, 14 mmol, 2.1 equiv.) was added portionwise to the reaction mixture at room temperature (caution: vigorous evolution of N_2 gas was observed). The reaction mixture was stirred at room temperature for an additional 2 h, after which the solvent was removed under reduced pressure. Saturated Na_2CO_3 (200 mL) was added slowly to the reaction mixture, which was stirred for 30 min until CO_2 evolution ceased. The reaction mixture was extracted with DCM (3 × 50 mL). The combined organic layer was washed with water (200 mL), brine (200 mL), dried over anhydrous Na_2SO_4, and concentrated using a rotary evaporator. The crude mixture was purified by flash column chromatography over silica gel (0–30% EtOAc/hexanes) to give **S1** as an orange solid (0.73 g, 45%). ^{1}H NMR (400 MHz, 298 K, $CDCl_3$) δ 7.22 (d, *J* = 8.0 Hz, 2H), 7.01 (d, *J* = 8.0 Hz, 2H), 6.92 (d, *J* = 8.0 Hz, 2H), 6.62 (d, *J* = 8.0 Hz, 2H), 1.63 (s, 6H). ^{13}C{^{1}H} NMR (101 MHz, 298 K, $(CD_3)_2SO$) δ 148.4, 144.1, 140.7, 137.2, 128.3, 127.7, 118.6, 115.1, 42.0, 31.0 ppm. HRMS (ESI) m/z: $[M+H]^+$ calcd for $C_{15}H_{17}N_4$ 253.1453; found 253.1454.

Compound S2. Compound **S2** was synthesized following a modified literature procedure.[7] Compound **S1** (0.20 g, 0.80 mmol, 1.0 equiv.) was dissolved in a 1,4-dioxane/water mixture (*v/v* = 2:1, 3.0 mL, 0.26 M) in a 25 mL round-bottom flask and cooled to 0 °C in a water ice bath. Thiophosgene (0.094 mL, 0.80 mmol, 1.2 equiv.) was added dropwise to the reaction mixture at 0 °C. The reaction mixture was warmed to room temperature and stirred for 2 h. The reaction progress was monitored by TLC. The reaction mixture was partitioned between DCM (30 mL) and saturated Na_2CO_3 (30 mL). The combined organic layer was washed with water (3 × 10 mL), brine (3 × 10 mL), dried over anhydrous Na_2SO_4, and concentrated using rotary evaporator. The crude reaction mixture was purified by silica gel column chromatography (0–40% EtOAc/hexanes) to afford a mixture of product **S2** and a small amount of unreacted **S1**. The crude product was washed with hexanes/DCM (*v/v* = 10:1, 5 × 1.0 mL) to yield **S2** as an off-white powder (0.39 g, 90%). ^{1}H NMR (400 MHz, 298 K, $(CD_3)_2SO$) δ 9.67 (s, 2H), 7.38 (d, *J* = 8.0 Hz, 4H), 7.27 (d, *J* = 8.0 Hz, 4H), 7.16 (d, *J* = 8.0 Hz, 4H), 7.04 (d, *J* = 8.0 Hz, 4H), 1.62 (s, 12H). ^{13}C{^{1}H} NMR (101 MHz, 298 K, $(CD_3)_2SO$) δ 179.4, 147.3, 146.0, 137.1, 136.7, 128.1, 126.4, 123.2, 118.7, 41.9, 30.3 ppm. HRMS (ESI) m/z: $[M+H]^+$ calcd for $C_{31}H_{31}N_8S$ 547.2392; found 547.2398.

Compound 1a. Compound **1a** was synthesized following a modified literature procedure.[8] An oven-dried 25 mL round-bottom flask was charged with **S2** (109 mg, 0.20 mmol, 1.0 equiv.), **S3** (107 mg, 0.60 mmol, 3.0 equiv.), and CuI (38 mg, 0.060 mmol, 0.3 equiv.). The flask was

evacuated and backfilled with N_2 (×3). MeCN (2.5 mL, 0.08 M) was added to the reaction mixture, followed by DIEA (0.10 mL, 0.4 mmol, 2 equiv.). The reaction mixture was stirred at room temperature for 48 h. The resulting precipitate was collected by filtration and washed with 2% of H_2O/acetone (5 × 1.0 mL). The crude solid was purified by column chromatography on neutral alumina, eluting with 5–50% MeOH/acetone containing 1% acetic acid as a modifier, to afford a slightly orange solid containing minor unidentified impurities (150 mg). Further purification was carried out by dissolving the crude solid in hot DCM/MeOH (v/v = 1:1, 1.0 mL), followed by slow addition of Et_2O at room temperature until the solution became cloudy. The mixture was stored at −20 °C for 16 h. The solid formed was collected by filtration and washed with diethyl ether (5 × 1.0 mL) to yield **1a** as an off-white powder (36 mg, 30% yield). ^{1}H NMR (400 MHz, 298 K, $(CD_3)_2SO$) δ 10.32 (s, 2H), 9.04 (s, 2H), 7.84 (d, *J* = 8.3 Hz, 4H), 7.48 (dd, *J* = 8.7, 2.4 Hz, 8H), 7.20 (d, *J* = 8.4 Hz, 4H), 4.74 (s, 4H), 3.10 (s, 18H), 1.70 (s, 12H). ^{13}C{^{1}H} NMR (101 MHz, 298 K, $(CD_3)_2SO$) δ 179.2, 151.5, 145.5, 137.3, 136.4, 134.1, 127.9, 126.4, 123.0, 120.2, 59.1, 51.9, 42.2, 30.2 ppm. HRMS (ESI) m/z: $[M]^{2+}$ calcd for $C_{43}H_{54}N_{10}S^{2+}$ 371.2122; found 371.2122.

Compound 1b. Compound **1b** was synthesized following a modified literature procedure.[7] A solution of 4,4′-(propane-2,2-diyl)dianiline (45 mg, 0.20 mmol, 1.0 equiv) in 1,4-dioxane/H_2O (v/v = 2:1, 1.2 mL, 0.20 M) was cooled to 0 °C. Thiophosgene (purity ~85%; 0.065 mL, 0.48 mmol, 2.4 equiv) was added dropwise. The reaction mixture was allowed to warm to room temperature and stirred for 2 h. Upon completion of the reaction as indicated by TLC, dichloromethane (30 mL) was added, and the mixture was washed sequentially with saturated aqueous $NaHCO_3$ (3 × 10 mL), water (3 × 10 mL), and brine (3 × 10 mL). The organic layer was dried over anhydrous Na_2SO_4, filtered, and concentrated under reduced pressure. The crude residue was dissolved in DCM (0.60 mL, 0.33 M), followed by the addition of pyridine (0.024 mL, 0.30 mmol, 1.5 equiv) and methylamine (2.0 M in THF; 0.40 mL, 0.80 mmol, 4.0 equiv). The reaction mixture was stirred at room temperature for 48 h, during which a precipitate formed. The solid was collected by filtration and washed with hexanes/DCM (*v*/*v* = 10:1, 5 × 1.0 mL) to afford an off-white powder (70 mg, quantitative yield). ^{1}H NMR (400 MHz, 298 K, $(CD_3)_2SO$) δ 9.48 (s, 2H), 7.62 (s, 2H), 7.26 (d, *J* = 8.0 Hz, 4H), 7.19 (d, *J* = 8.0 Hz, 4H), 2.90 (d, *J* = 4.0 Hz, 6H), 1.62 (s, 6H). ^{13}C{^{1}H} NMR (101 MHz, 298 K, $(CD_3)_2SO$) δ 180.9, 146.0, 136.6, 128.0, 126.7, 125.6, 122.8, 41.7, 31.2, 30.4 ppm. HRMS (ESI) m/z: $[M+H]^+$ calcd for $C_{19}H_{25}N_4S_2$ 373.1516; found 373.1516.

Compound 1d. Compound **1d** was synthesized following a modified literature procedure.[7] A solution of 4,4'-methylenedianiline (345 mg, 1.0 mmol, 1.0 equiv) in 1,4-dioxane/H_2O (v/v = 2:1, 5.0 mL, 0.20 M) was cooled to 0 °C in an ice–water bath. Thiophosgene (purity ~85%; 0.22 mL, 2.4 mmol, 2.4 equiv) was added dropwise at 0 °C. The reaction mixture was allowed to warm to room temperature and stirred for 2 h. Upon completion (as monitored by TLC), dichloromethane (30 mL) was added, and the mixture was washed sequentially with saturated aqueous $NaHCO_3$ (3 × 10 mL), water (3 × 10 mL), and brine (3 × 10 mL). The organic layer was dried over anhydrous Na_2SO_4, filtered, and concentrated under reduced pressure. The crude residue was dissolved in DCM (3.0 mL, 0.33 M), followed by the addition of pyridine (0.081 mL, 1.0 mmol, 1.0 equiv) and methylamine (2.0 M in THF; 2.0 mL, 4.0 mmol, 4.0 equiv). The reaction mixture was stirred at room temperature for 48 h, during which a precipitate formed. The solid was collected by filtration and washed with hexanes/DCM (*v*/*v* = 10:1, 5 × 1.0 mL) to afford an off-white powder (352 mg, quantitative yield). ^{1}H NMR (600 MHz, 298 K, CD_3OD) δ 7.20 – 7.12 (m, 8H), 3.91 (s, 2H), 2.97 (s, 6H). ^{13}C{^{1}H} NMR (151 MHz, 298 K, CD_3OD) δ 182.6, 140.2, 131.1, 130.7, 126.6, 125.9, 41.7, 32.0 ppm. HRMS (ESI) m/z: $[M+H]^+$ calcd for $C_{17}H_{21}N_4S_2$ 345.1208; found 345.1218.

Compound S4. Compound **S4** was synthesized following a modified literature procedure.[11] Dansyl chloride (0.54 g, 2.0 mmol, 1.0 equiv.) was dissolved in dry DCM in a 6-dram vial at room temperature. 2-Azidoethanamine (0.35 g, 4.0 mmol, 2.0 equiv.) and Et_3N (0.56 mL, 4.0 mmol, 2.0 equiv.) were added to the reaction mixture *via* syringe under continuous flow of N_2. The reaction mixture was stirred for 16 h at room temperature and was concentrated using a rotary evaporator. The crude mixture was purified by flash column chromatography over silica gel (0–35% EtOAc/hexanes) to give **S4** as a yellow oil (0.31 g, 49%). ^{1}H NMR (600 MHz, 298 K, $CDCl_3$) δ 8.57 (d, *J* = 8.5 Hz, 1H), 8.29 – 8.22 (m, 2H), 7.59 (dd, *J* = 8.6, 7.5 Hz, 1H), 7.53 (dd, *J* = 8.5, 7.3 Hz, 1H), 7.21 (d, *J* = 7.5 Hz, 1H), 5.08 (t, *J* = 6.3 Hz, 1H), 3.30 (t, *J* = 5.7 Hz, 2H), 3.06 (q, *J* = 6.0 Hz, 2H), 2.89 (s, 6H). ^{13}C{^{1}H} NMR (151 MHz, 298 K, $CDCl_3$) δ 152.2, 134.6, 130.9, 130.1, 129.8, 129.6, 128.8, 123.3, 118.6, 115.5, 51.1, 45.6, 42.5 ppm. HRMS (ESI) m/z: $[M+H]^+$ calcd for $C_{14}H_{18}N_5O_2S^+$ 320.1176; found 320.1185.

Compound S6. Compound **S6** was synthesized following a modified literature procedure.[12] Compound **S4** (0.32 g, 1.0 mmol, 1.0 equiv.), CuBr (0.14 g, 1.0 mmol, 1.0 equiv.), and compound **S5** (0.27 g, 1.0 mmol, 1.0 equiv.) were dissolved in dry DMF in a 25 mL round bottom flask under continuous flow of N_2 at room temperature. PMDETA (0.20 mL, 1.0 mmol, 1.0 equiv.) was added

dropwise to the reaction at room temperature. Upon completion of the reaction as indicated by TLC, water (20 mL) was added to the mixture. The crude mixture was extracted with EtOAc (5 × 20 mL). The organic layer was combined and washed sequentially with water (3 × 100 mL) and brine (3 × 100 mL), and was concentrated using a rotary evaporator to give **S6** as a yellow powder (0.47 g, 84%). The resulting powder was used directly in the subsequent step without further purification. A portion of the crude mixture was purified by flash column chromatography over silica gel (40–90% EtOAc/hexanes) to get good characterization data. ^{1}H NMR (600 MHz, 298 K, $CDCl_3$) δ 8.56 (d, *J* = 8.5 Hz, 1H), 8.23 (d, *J* = 7.2 Hz, 1H), 8.18 (d, *J* = 8.7 Hz, 1H), 7.51 (q, *J* = 8.7 Hz, 2H), 7.45 (s, 1H), 7.16 (d, *J* = 7.6 Hz, 1H), 7.12 (d, *J* = 8.7 Hz, 2H), 7.05 (d, *J* = 8.7 Hz, 2H), 6.83 (d, *J* = 8.8 Hz, 2H), 6.72 (d, *J* = 8.6 Hz, 2H), 5.68 (s, 1H), 5.57 (s, 1H), 5.05 (s, 2H), 4.36 (t, *J* = 5.6 Hz, 2H), 3.44 (q, *J* = 5.9 Hz, 2H), 2.87 (s, 6H), 1.61 (s, 6H). ^{13}C{^{1}H} NMR (151 MHz, 298 K, $CDCl_3$) δ 156.1, 153.7, 144.3, 144.0, 143.1, 134.4, 131.0, 129.7, 129.5, 128.9, 128.0, 127.9, 124.2, 123.4, 115.6, 114.9, 114.4, 114.3, 61.8, 50.4, 45.6, 42.8, 41.8, 31.1. HRMS (ESI) m/z: $[M+H]^+$ calcd for $C_{32}H_{36}N_5O_4S^+$ 586.2483; found 586.2463.

Compound 5. Compound **5** was synthesized following a modified literature procedure.[13] A 10-mL glass seal tube was charged with **S7** (123 mg, 0.40 mmol, 1.0 equiv), followed by addition of MeCN (1.6 mL, 0.25 M) and MeI (0.25 mL, 4.0 mmol, 10 equiv) at room temperature. The reaction was sealed and stirred at 84 °C for 5 h. Upon completion of the reaction as indicated by TLC, the reaction mixture was transferred to a 6-dram vial and concentrated under reduced pressure. The crude residue was dissolved in DCM (1.6 mL, 0.25 M), followed by the addition of **S6** (234 mg, 0.4 mmol, 1.0 equiv). The reaction mixture was stirred at at room temperature for 5 h. Upon completion of the reaction, dichloromethane (10 mL) was added, and the mixture was washed sequentially with water (3 × 10 mL), and brine (3 × 10 mL). The organic layer was dried over anhydrous Na_2SO_4, filtered, and concentrated under reduced pressure. The crude mixture was purified by silica gel column chromatography (0–40% EtOAc/hexanes) to afford compound **5** as a light yellow powder (170 mg, 55% yield). ^{1}H NMR (600 MHz, 298 K, $CDCl_3$) δ 8.58 (s, 1H), 8.25 (dd, *J* = 7.2, 1.3 Hz, 1H), 8.20 (d, *J* = 8.7 Hz, 1H), 7.53 (dt, *J* = 8.7, 6.2 Hz, 2H), 7.45 (s, 1H), 7.30 – 7.26 (m, 3H), 7.25 – 7.22 (m, 5H), 7.20 – 7.13 (m, 8H), 6.87 (d, *J* = 8.9 Hz, 2H), 5.59 (s, 1H), 5.08 (s, 2H), 4.37 (t, *J* = 5.6 Hz, 2H), 3.45 (q, *J* = 5.9 Hz, 2H), 2.89 (s, 6H), 1.68 (s, 6H), 1.66 (s, 6H). ^{13}C{^{1}H} NMR (151 MHz, 298 K, $CDCl_3$) δ 156.3, 152.4, 150.3, 149.0, 149.0, 149.0, 148.9, 144.3, 143.2, 134.4, 131.0, 130.0, 129.7, 129.5, 128.9, 128.2, 128.1, 128.0, 126.9, 125.9,

124.1, 123.3, 120.3, 118.6, 115.6, 114.4, 50.3, 45.6, 42.9, 42.8, 42.3, 31.1, 30.9. HRMS (ESI) m/z: $[M+H]^+$ calcd for $C_{48}H_{50}N_5O_6S^+$ 824.3476; found 824.3483.

Characterization of MINPs

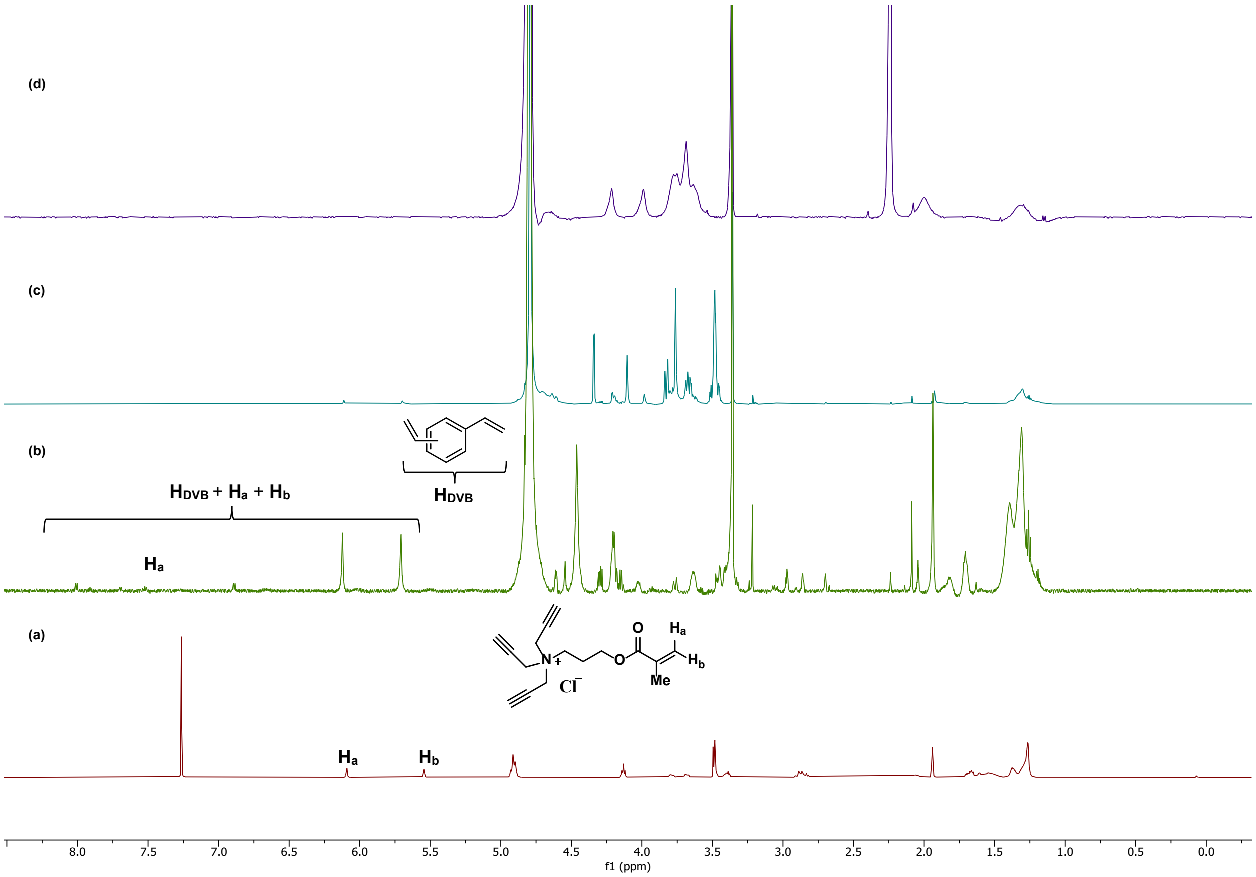

Figure S1. Surface-cross-linking and the core-cross-linking of the micelles were monitored by ^{1}H NMR spectroscopy. ^{1}H NMR spectra of (a) surfactant **2** in $CDCl_3$, (b) typical alkynyl surface cross-linked micelle in D_2O, (c) typical surface-functionalized micelle in D_2O, (d) typical MINP(**1a**/**4a**) after washing and re-dissolving in D_2O.

Dynamic Light Scattering

The particle size of MINP was determined on a Malvern Zetasizer Nano ZS using the Zetasizer software according to the Stokes-Einstein equation (1). The volume of a spherical nanoparticle (V_{D_h}) was calculated from equation (2). Assuming a density of 1.37 g/cm^3 (the density of protein), the molecular weight of the particle can be calculated using equation (3).[3] A nanoparticle with a hydrodynamic diameter of 4.87 nm has a calculated molecular weight of 50 kDa, which was used in making MINP solution.

$$D_h = \frac{k_B T}{6\pi\eta D_t} \qquad (1)$$

in which D_h is the hydrodynamic diameter, D_t the translational diffusion coefficient measured by dynamic light scattering, T the temperature, k_B the Boltzmann's constant, and η is dynamic viscosity of water (0.890 cP at 298 K).

$$V_{D_h} = \frac{4\pi}{3}\left(\frac{D_h}{2}\right)^3 \qquad (2)$$

$$\text{Mw in dalton} = \left(\frac{D_h}{0.132}\right)^3 \qquad (3)$$

in which D_h is the hydrodynamic diameter in nm.

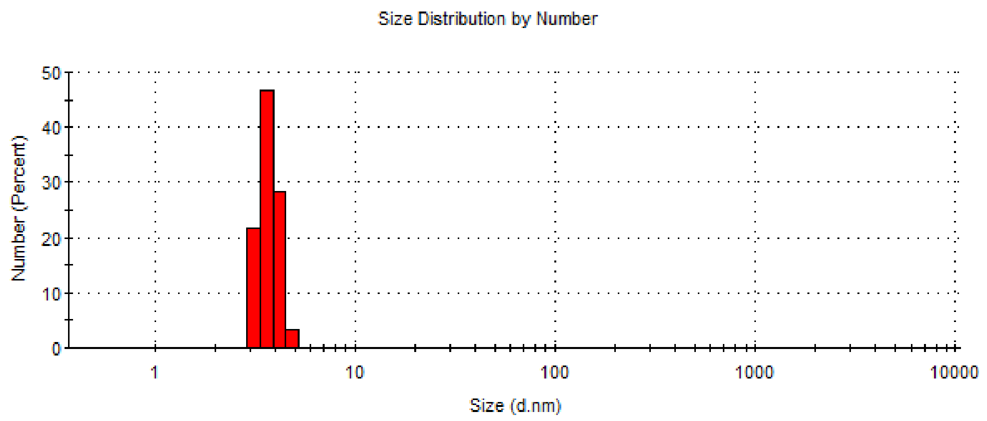

Figure S2. Distribution of the hydrodynamic diameters of the nanoparticles in water as determined by DLS for alkynyl-SCM (surface-cross-linked micelle). D = 3.77 ± 0.25 nm.

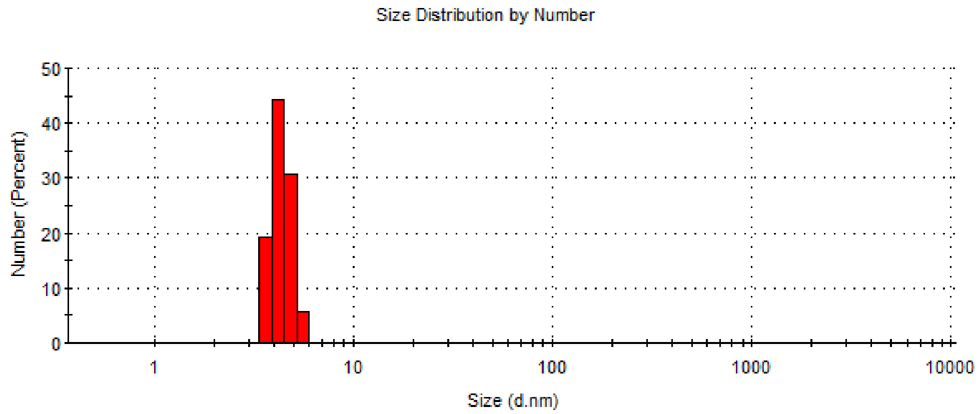

Figure S3. Distribution of the hydrodynamic diameters of the nanoparticles in water as determined by DLS for surface functionalized SCM (surface-cross-linked micelle). D = 4.46 ± 0.33 nm.

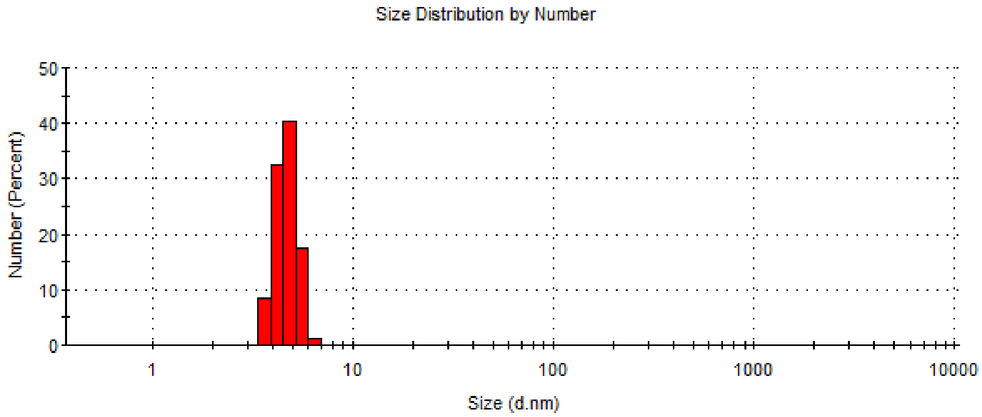

Figure S4. Distribution of the hydrodynamic diameters of the nanoparticles in water as determined by DLS for MINP(**1a**/**4a**). D = 4.83 ± 0.37 nm.

(a)

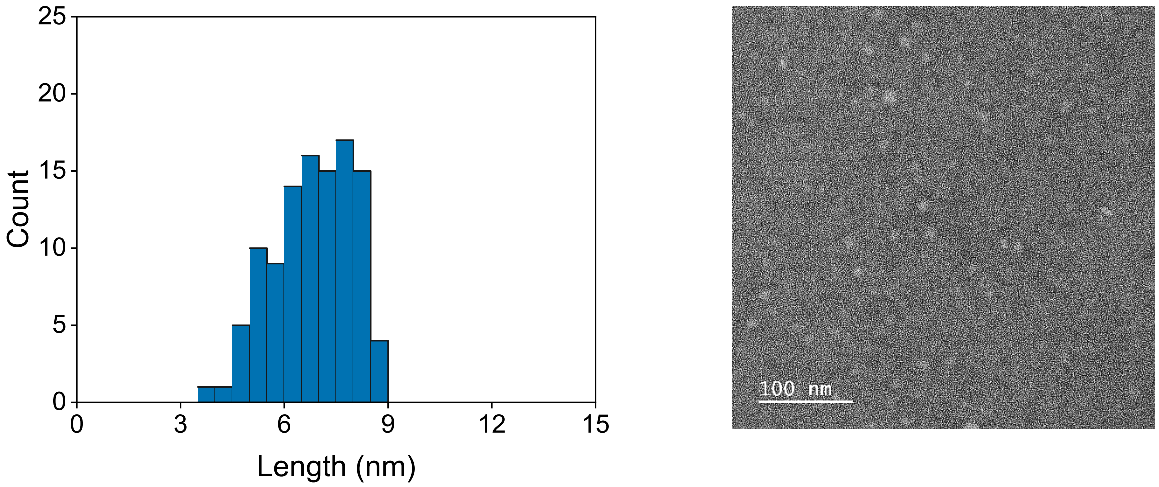

(b)

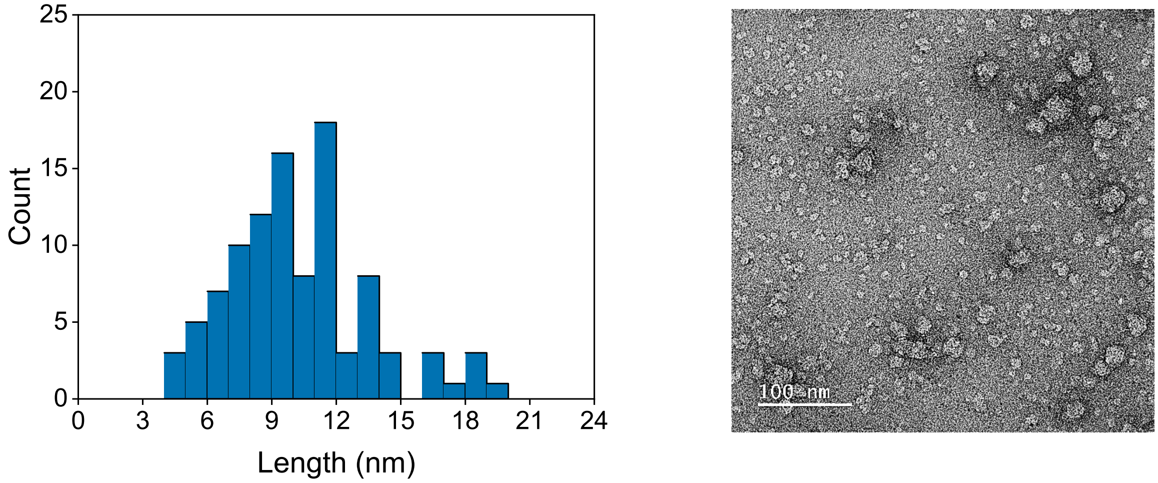

Figure S5. Conventional bright-field TEM image of typical MINPs (scale bar = 100 nm) for (a) 0.20 μM, (b) 2.0 μM. **General procedure**: A microsyringe was used to load one small drop (~1 μL) of the above solution onto a TEM copper grid covered with carbon film. The sample was left to form a thin layer, and then one small drop (~1 μL) of 2% uranyl acetate solution was loaded on the grid for the negative staining. The sample was left to dry and analyzed on a 200kV JEOL 2100 scanning/transmission electron microscope (STEM). **Note**: More MINP aggregation occurred at higher concentration.

Zeta potential

(a)

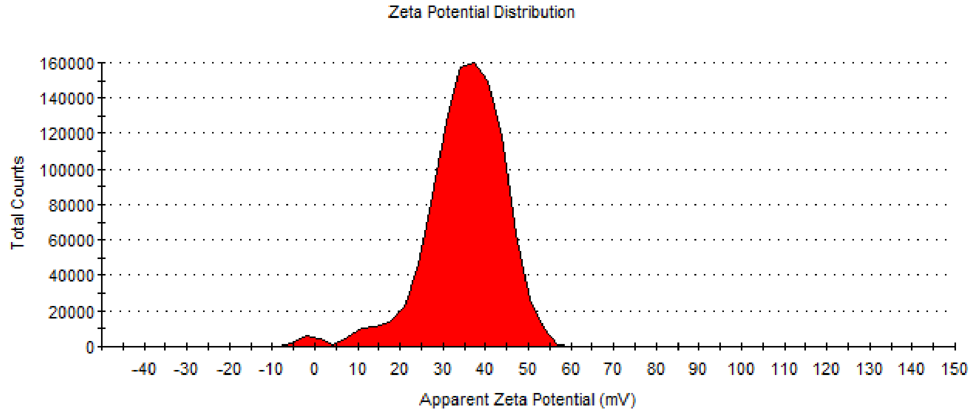

(b)

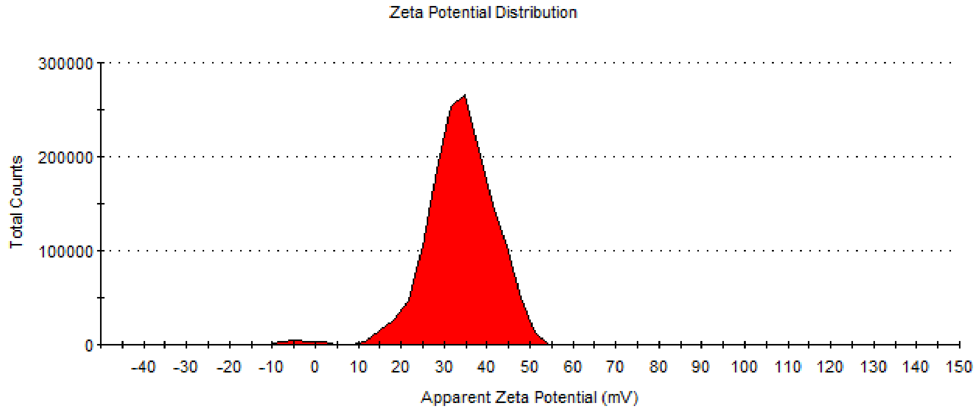

(c)

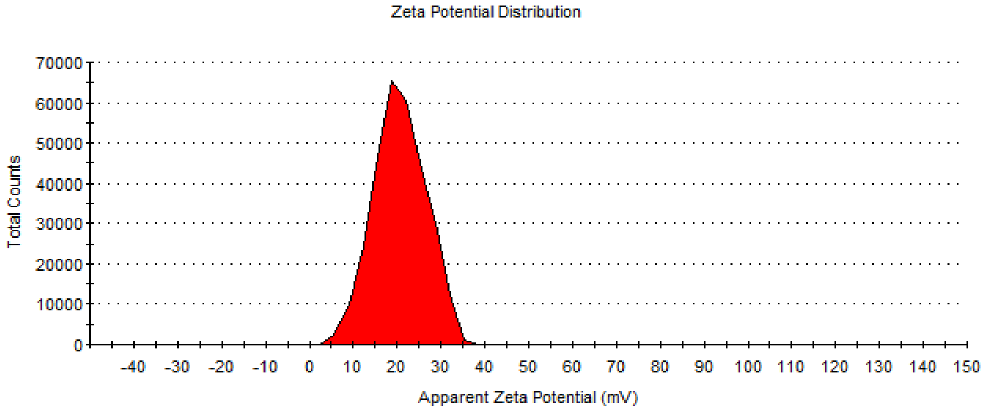

(d)

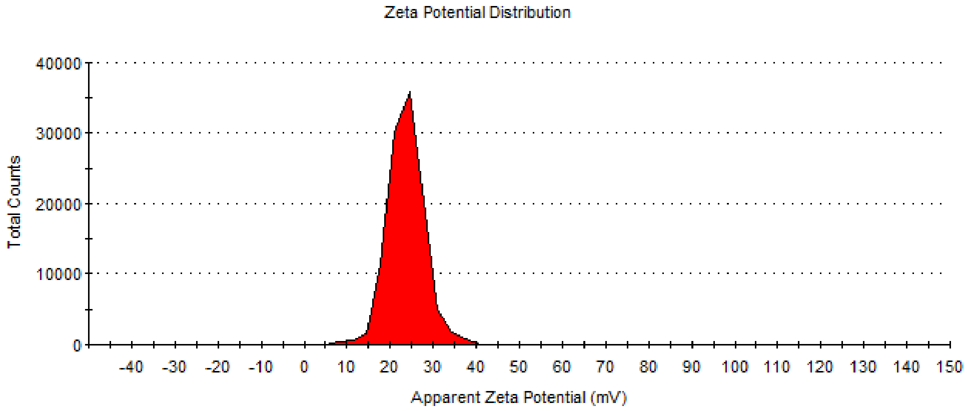

Figure S6. Zeta potential (a) for MINP(**1a**/**4a**), (b) for MINP(**1a**/**4b**), (c) for MINP(**1b**/**4a**) and (d) for MINP(**1b**/**4b**). The mean zeta potential values are 35.1 mV, 20.6 mV, 33.7 mV, 20.9 mV, respectively.

Determination of binding constant for compound 5 using fluorescence titration with different nanoparticles

In general, a stock solution (200 μM) of the appropriate MINP or NINP was prepared in water. An average M_w of 50 kDa was used for making stock solutions. Aliquots (2.0 μL) of the appropriate MINP or NINP stock solution were added to 2.00 mL of compound **5** (1.0 μM) in water. After each addition, the sample was allowed to sit for 1 min at room temperature before the fluorescence spectrum was recorded. The excitation wavelength (λ_{ex}) was 335 nm, and the emission wavelength (λ_{em}) was 493 nm. The excitation slit width was 10 nm, and the emission slit width was 5 nm. The binding constant was obtained by nonlinear least squares fitting in OriginPro of the emission intensity to a 1:1 binding isotherm.

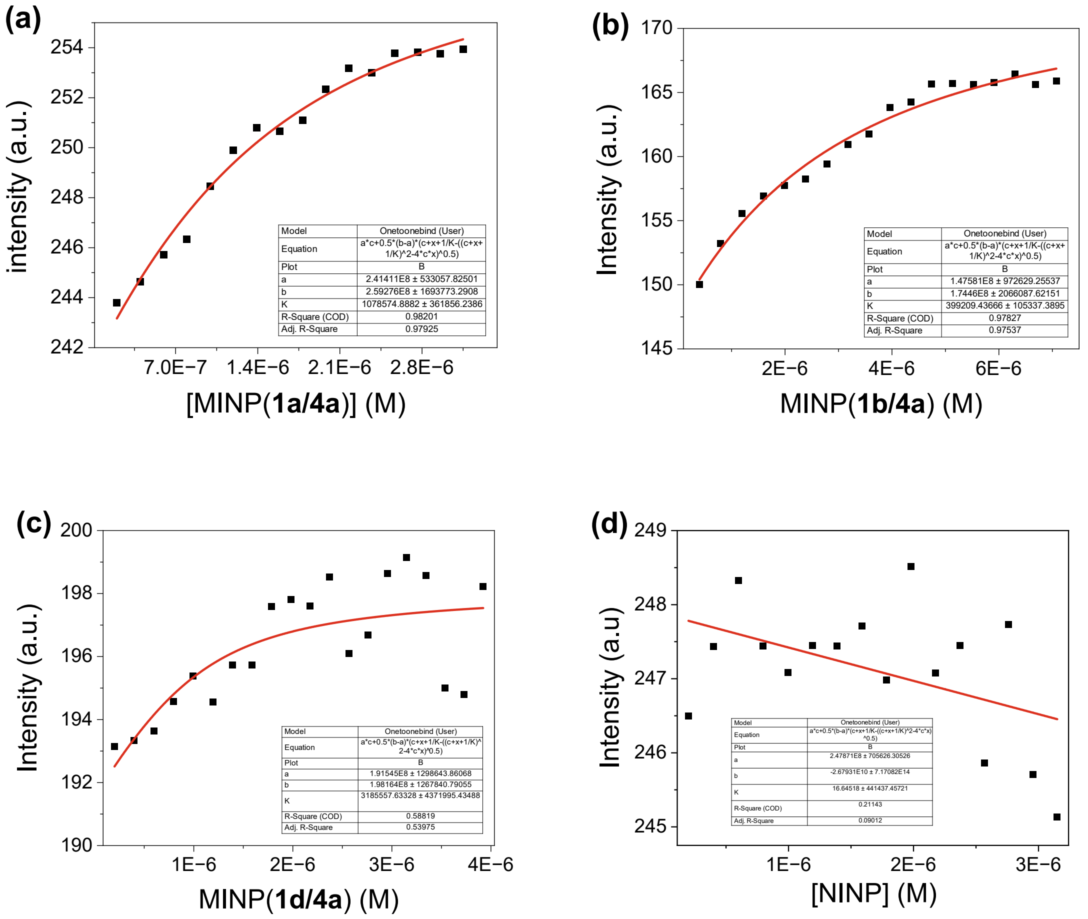

Figure S7. Fluorescence titration curves obtained at 298 K in water for the binding of **5** with (a) MINP (**1a/4a**), (b) MINP (**1b/4a**), (c) MINP (**1d/4a**), and (d) NINP. The data correspond to entries 1–4 in Table 2.

Isothermal Calorimetry (ITC) experiments

Generally, a solution of an appropriate template (**1a** or **1b**) in 5% DMSO in water (*v/v*) at 298.15 K was injected in equal steps into 1.43 mL of the corresponding nanozymes in the same solution. The top panel shows the raw calorimetric data. The area under each peak represents the amount of heat generated at each injection and is plotted as a function of the molar ratio of NP to guest. The smooth, solid line represents the best fit to the experimental data for the sequential binding of the N-binding site on the NP. The heat of dilution for the guest, obtained by titration beyond the saturation point, was subtracted from the heat released during binding. Binding parameters were auto-generated after curve fitting using Microcal Origin 7.

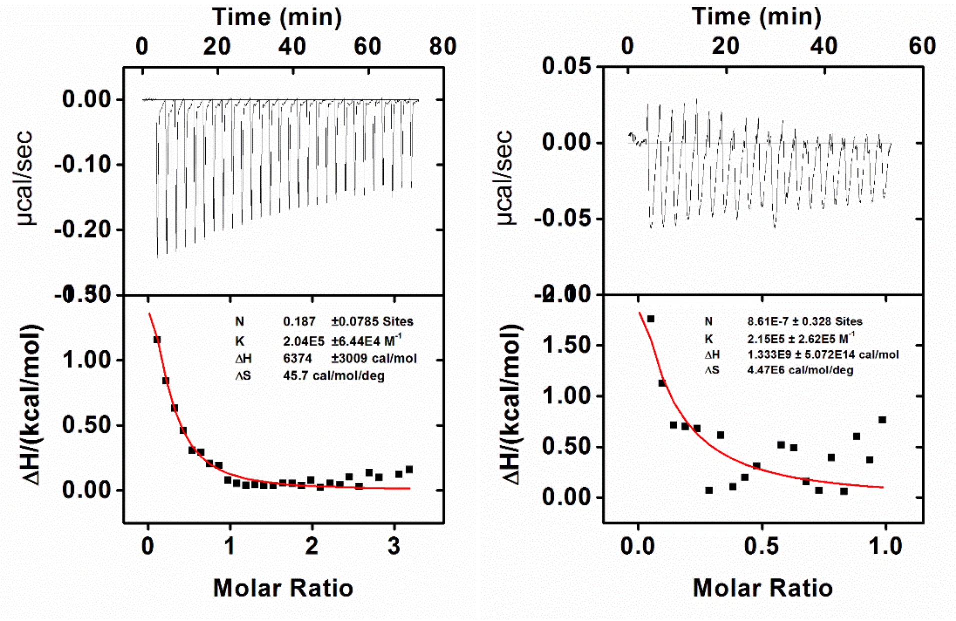

Figure S8. ITC titration curves obtained at 298 K for MINP(**1a**/**4a**) titrated with template **1a** in DMSO:H_2O (5% v/v). The data corresponds to entry 1 in Table 1.

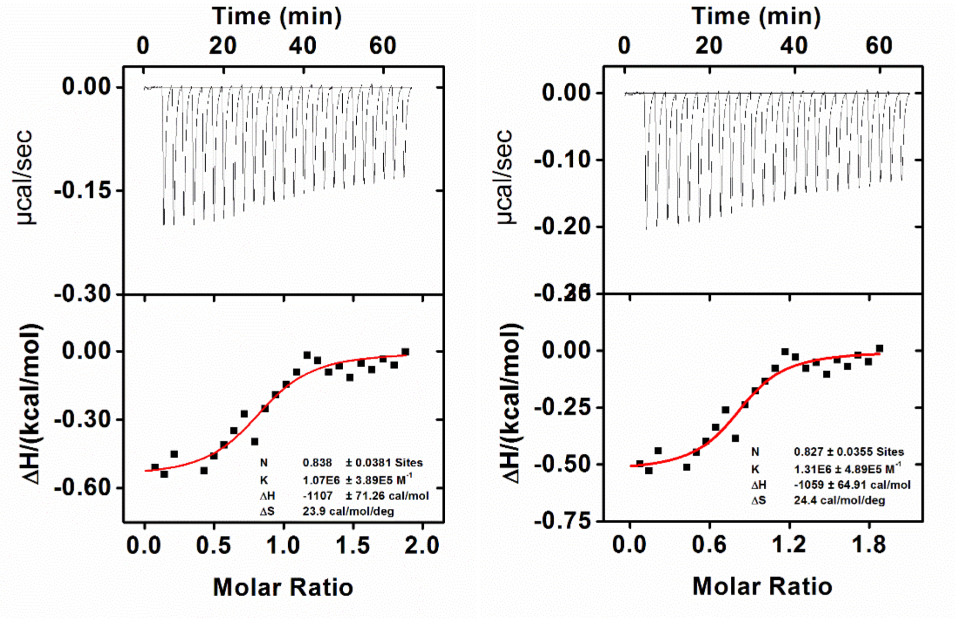

Figure S9. ITC titration curves obtained at 298 K for MINP(**1b**/**4a**) titrated with template **1b** in DMSO:H_2O (5% v/v). The data corresponds to entry 2 in Table 1.

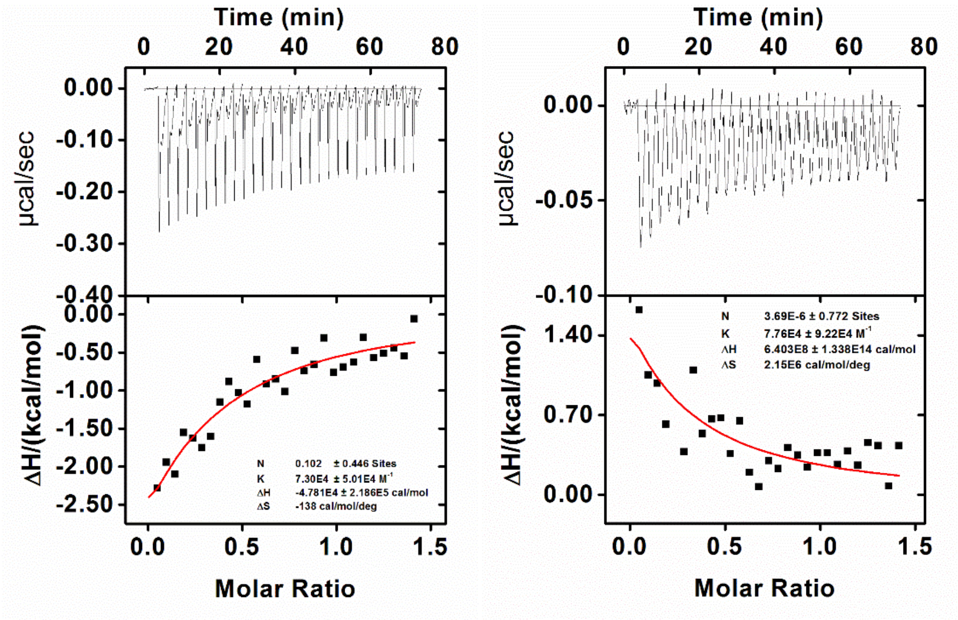

Figure S10. ITC titration curves obtained at 298 K for NINP titrated with template **1a** in DMSO:H_2O (5% v/v). The data corresponds to entry 3 in Table 1.

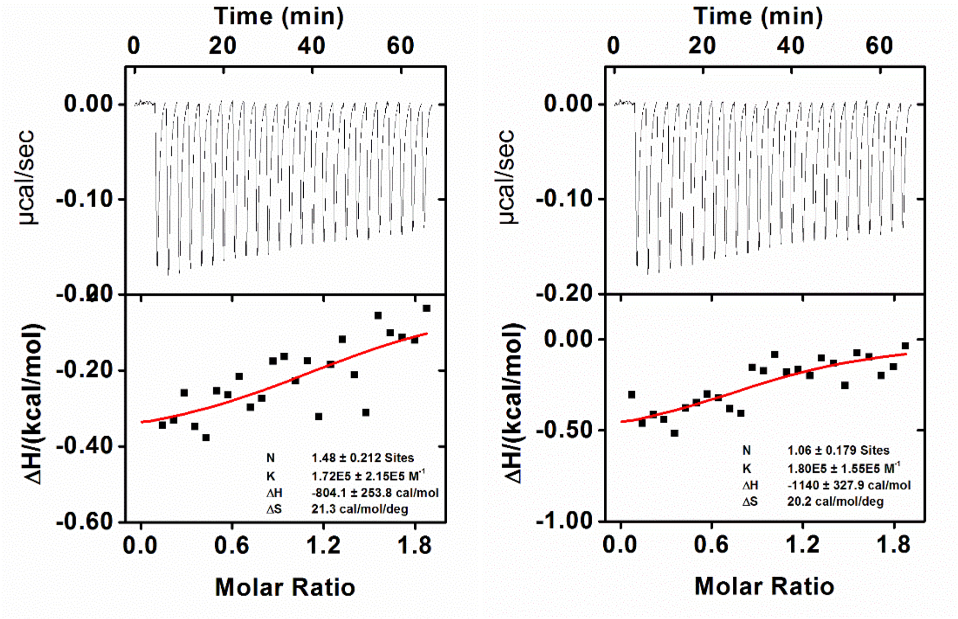

Figure S11. ITC titration curves obtained at 298 K for NINP titrated with template **1b** in DMSO:H_2O (5% v/v). The data corresponds to entry 4 in Table 1.

Study of degradation of bisphenol A polycarbonate (BPA-PC)

Mass spectrum analysis

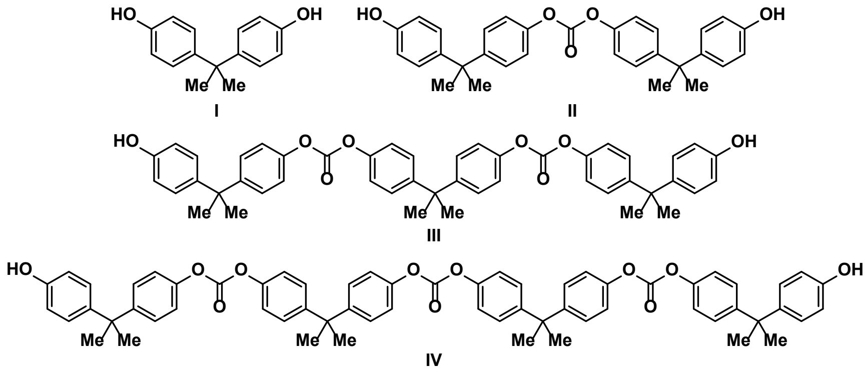

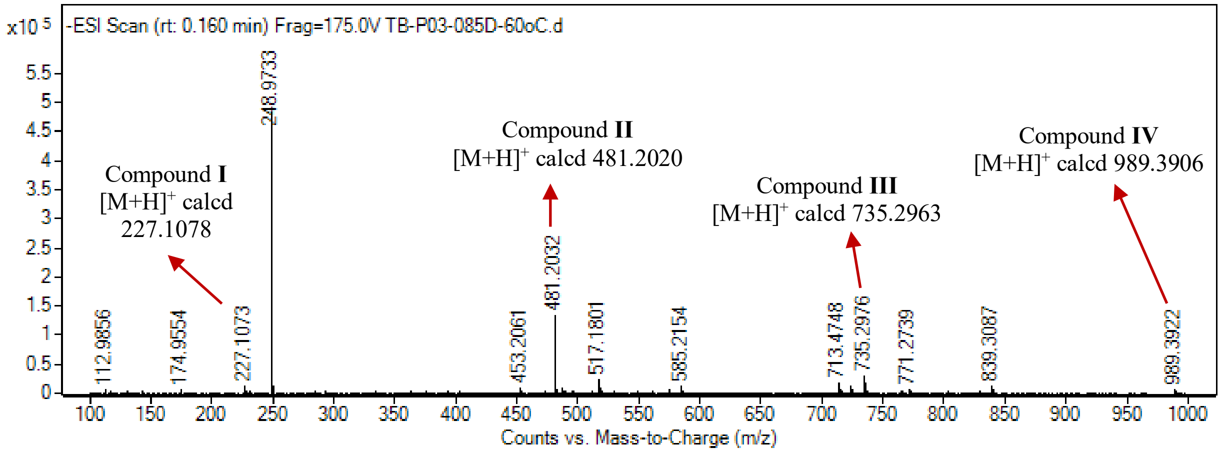

Figure S12. Mass spectrum of degradation of BPA-PC catalyzed by MINP(**1a**/**4a**) in 10 mM CHES buffer pH = 10.0 at 60 °C for 24 h. [MINP(**1a**/**4a**)] = 50 μM.

Quantitative analysis

Table S1. Quantitative result of BPA-PC degradation of MINP (**1b**) under standard catalytic condition.[a]

Remaining BPA-PC (%)	Oligomers (%)	BPA (%)	Mass balance	Note
89.6 ± 0.2	5.6 ± 0.5	0.47 ± 0.04	96%	~4% of lower M_w BPA-PC overlapped in ^{1}H-NMR

[a] Polymer degradation was performed in duplicates at 60 °C for 24 h in a 3:1 mixture of pH 10 CHES buffer and organic cosolvents (DMSO + MeOH). [MINP(**1b**)] = 50 μM.

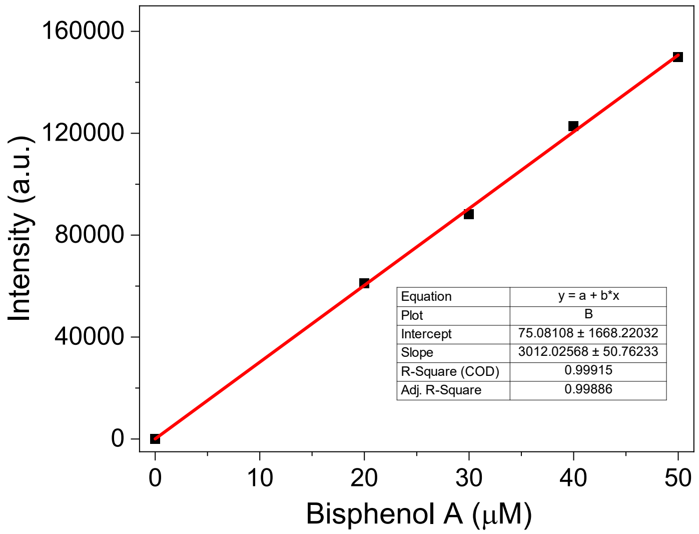

Figure S13. Calibration curve of bisphenol A.

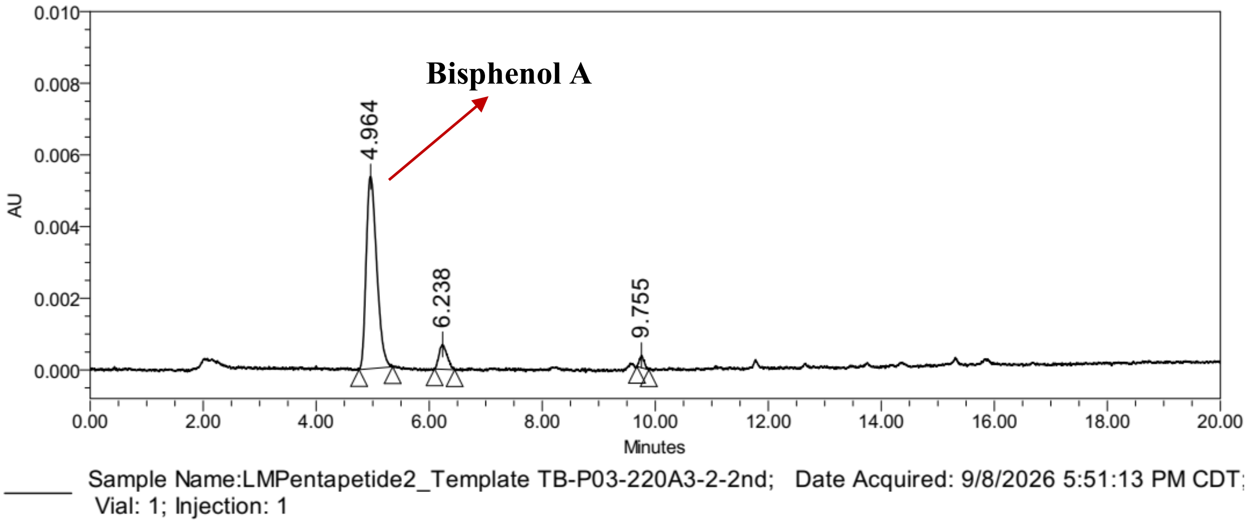

Figure S14. HPLC analysis of BPA-PC degradation 10 mM CHES buffer pH = 10.0 at 60 °C for 24 h. [MINP(**1b**)] = 50 μM.

Gel permeation chromatography analysis

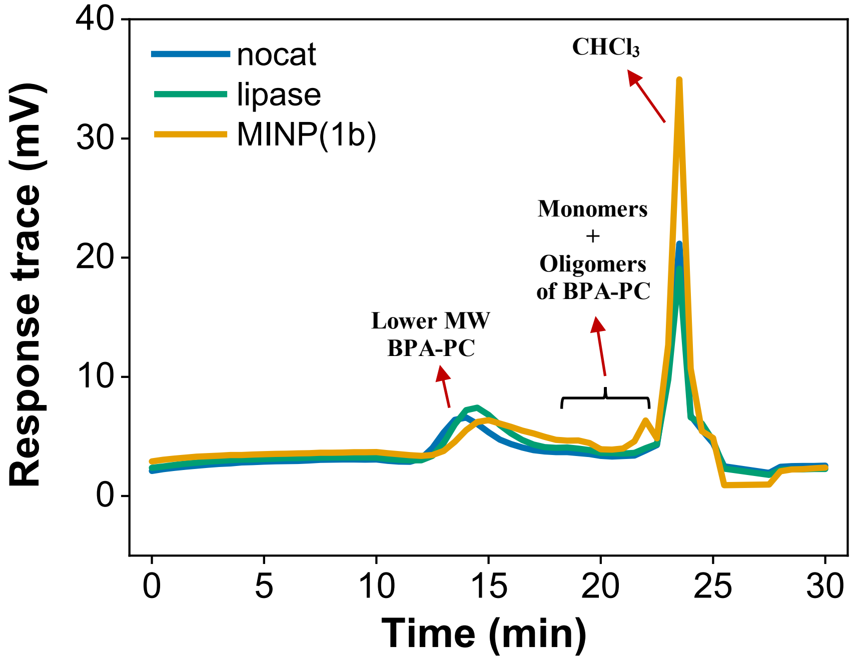

Figure S15. Gel permeation chromatography of BPA-PC degradation in 10 mM CHES buffer pH = 10.0 at 60 °C for 7 days. [MINP(**1b**)] = 50 µM, m_{lipase} = 5.0 mg.

^1H and $^{13}C\{^1H\}$ NMR spectra of key compounds

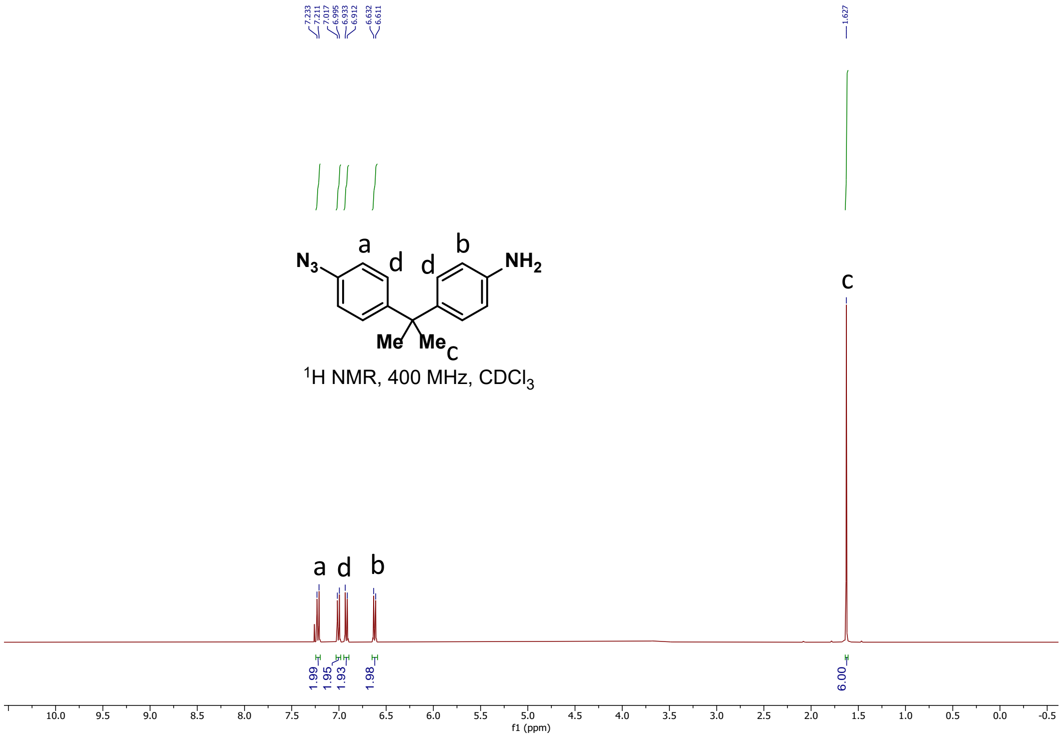

Figure S16. ^{1}H NMR spectrum of compound **S1**

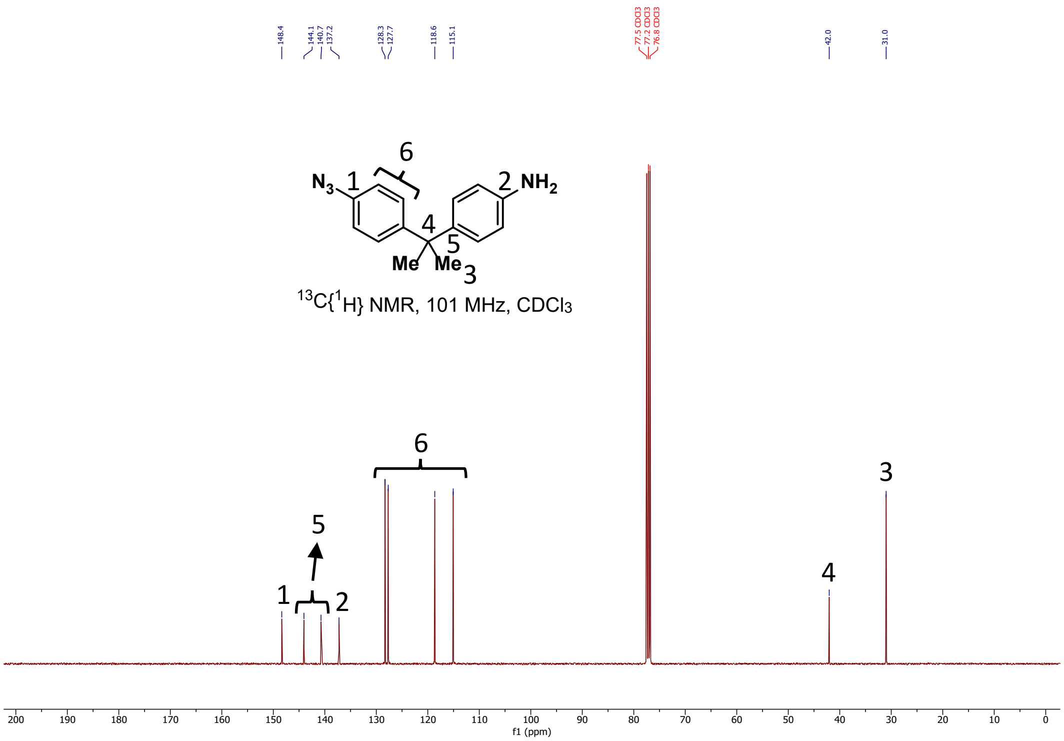

Figure S17. ^{13}C NMR spectrum of compound **S1**

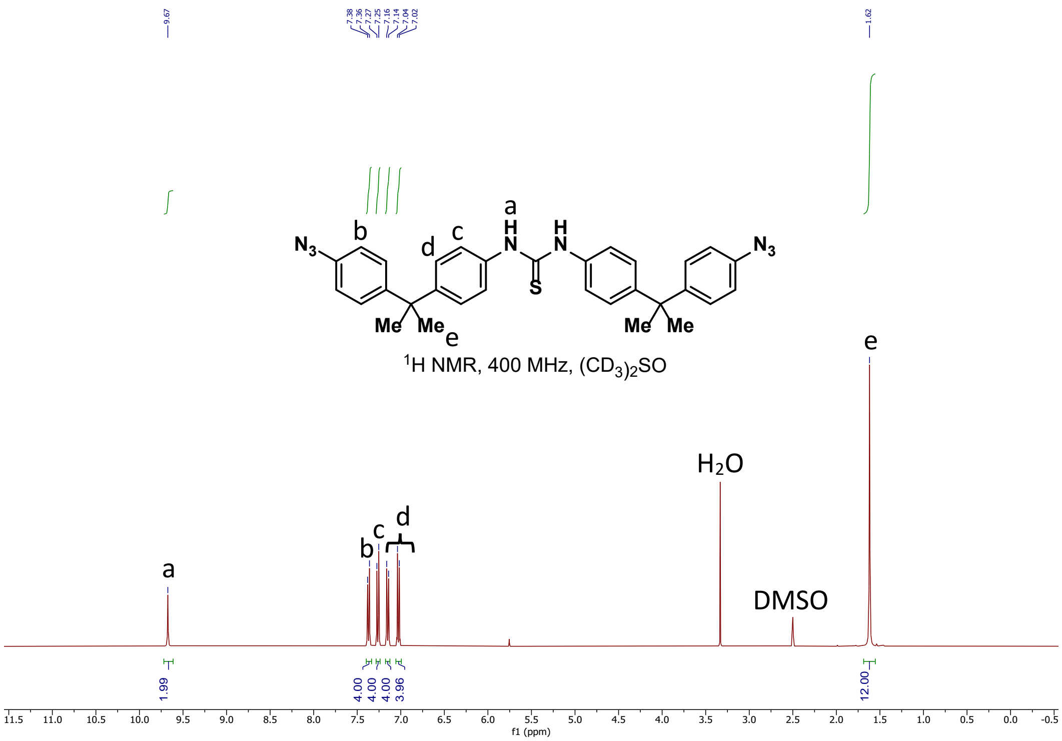

Figure S18. ^{1}H NMR spectrum of compound **S2**

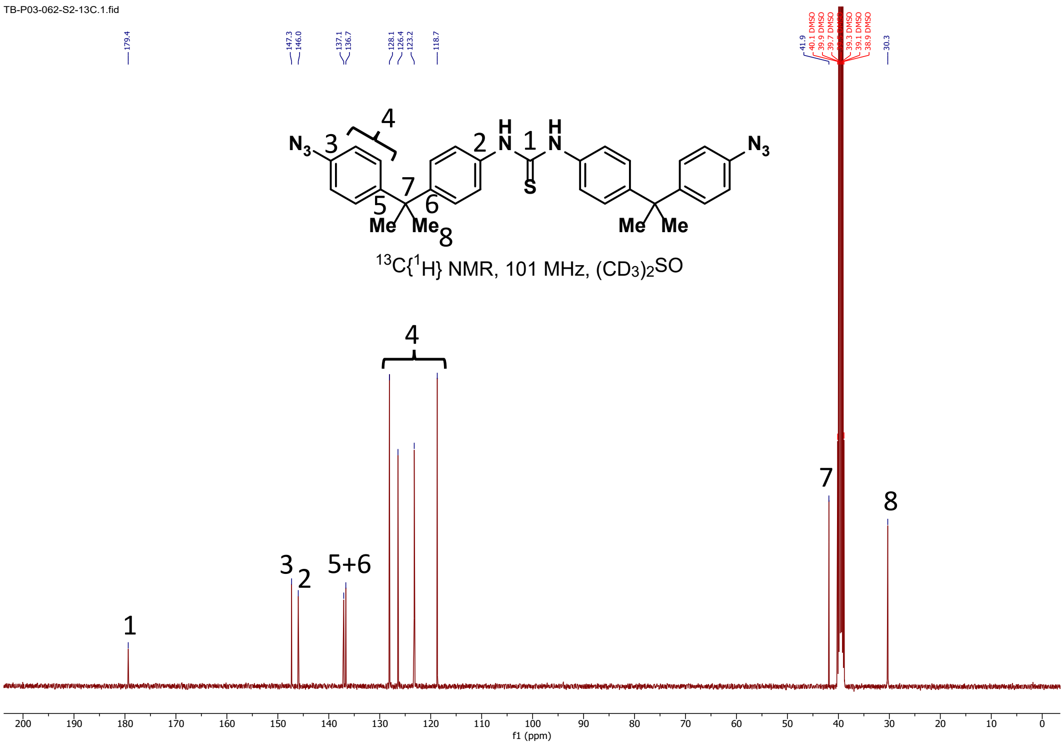

Figure S19. ^{13}C NMR spectrum of compound **S2**

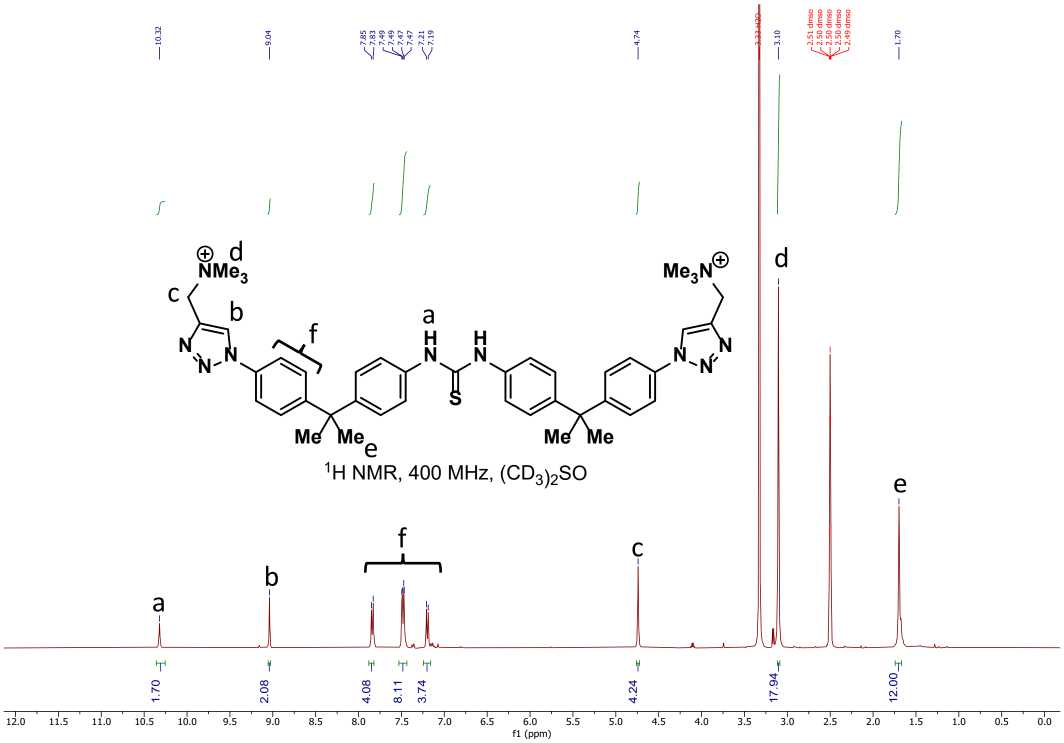

Figure S20. ^{1}H NMR spectrum of compound **1a**

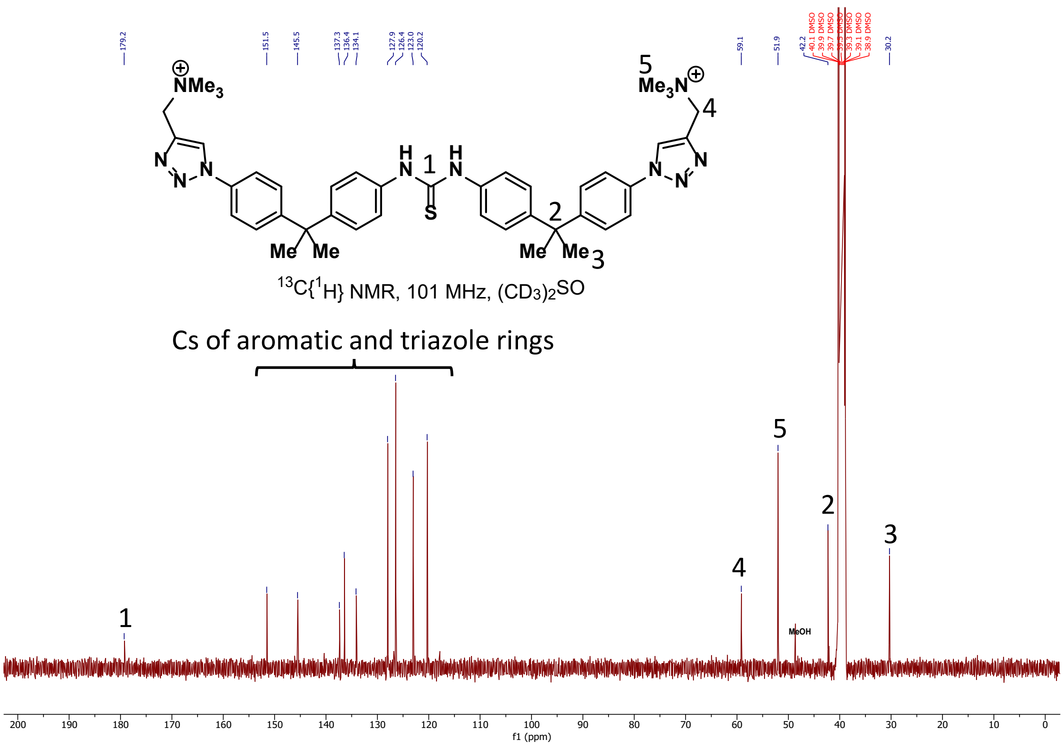

Figure S21. ^{13}C NMR spectrum of compound **1a**

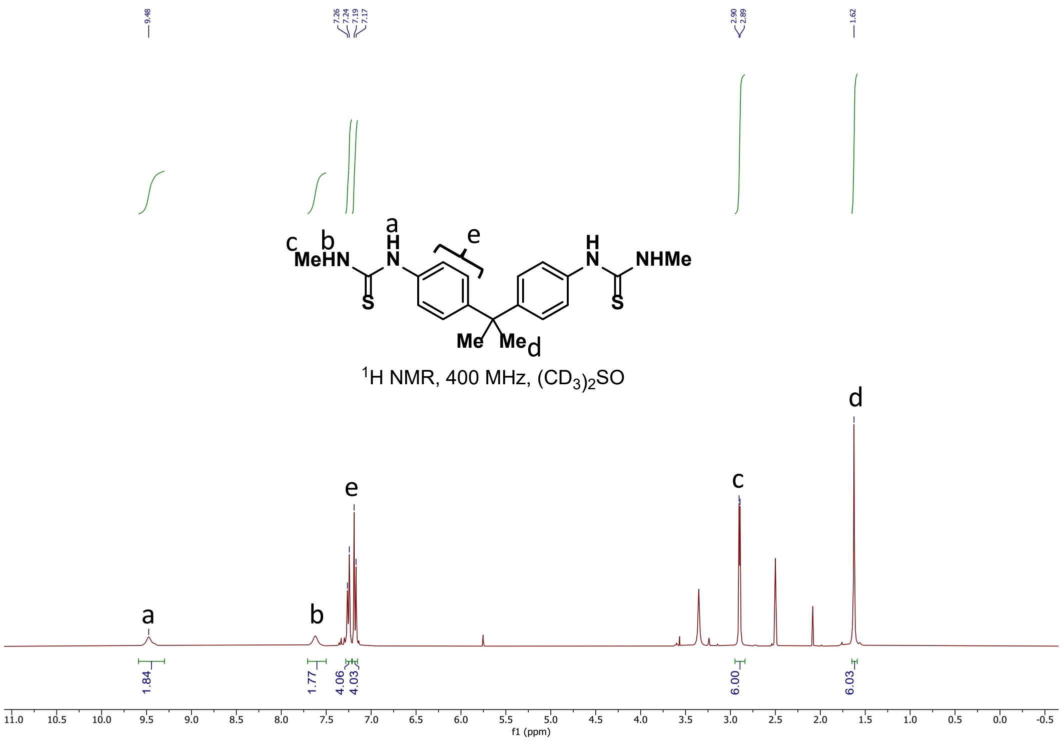

Figure S22. ^{1}H NMR spectrum of compound **1b**

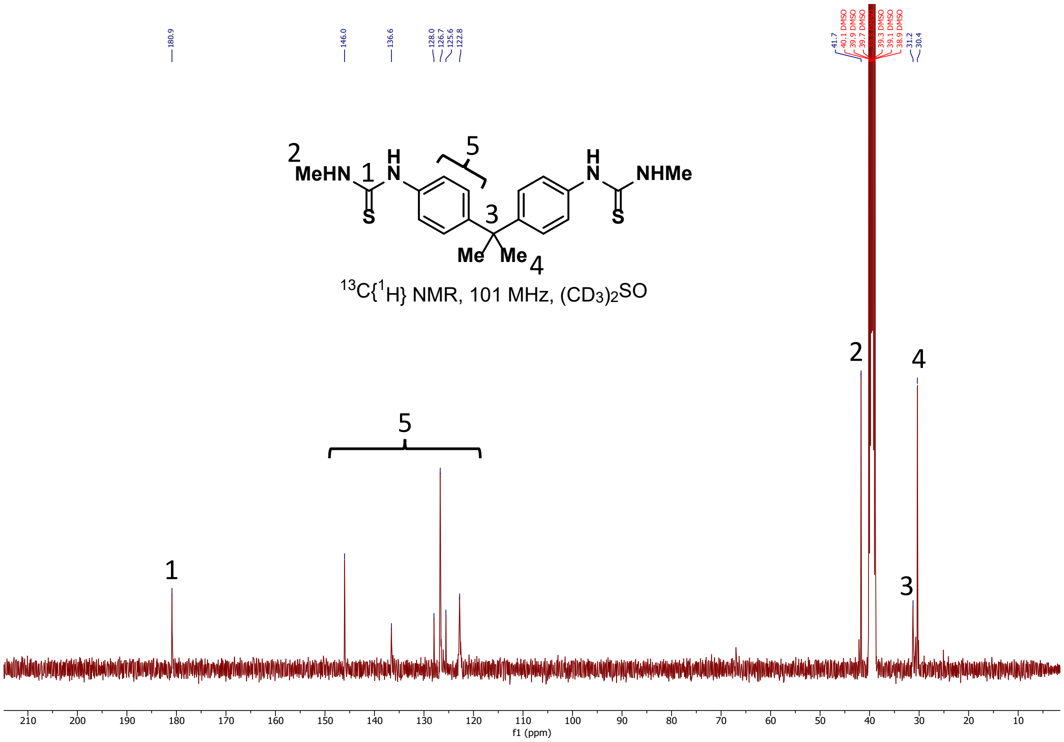

Figure S23. ^{13}C NMR spectrum of compound **1b**

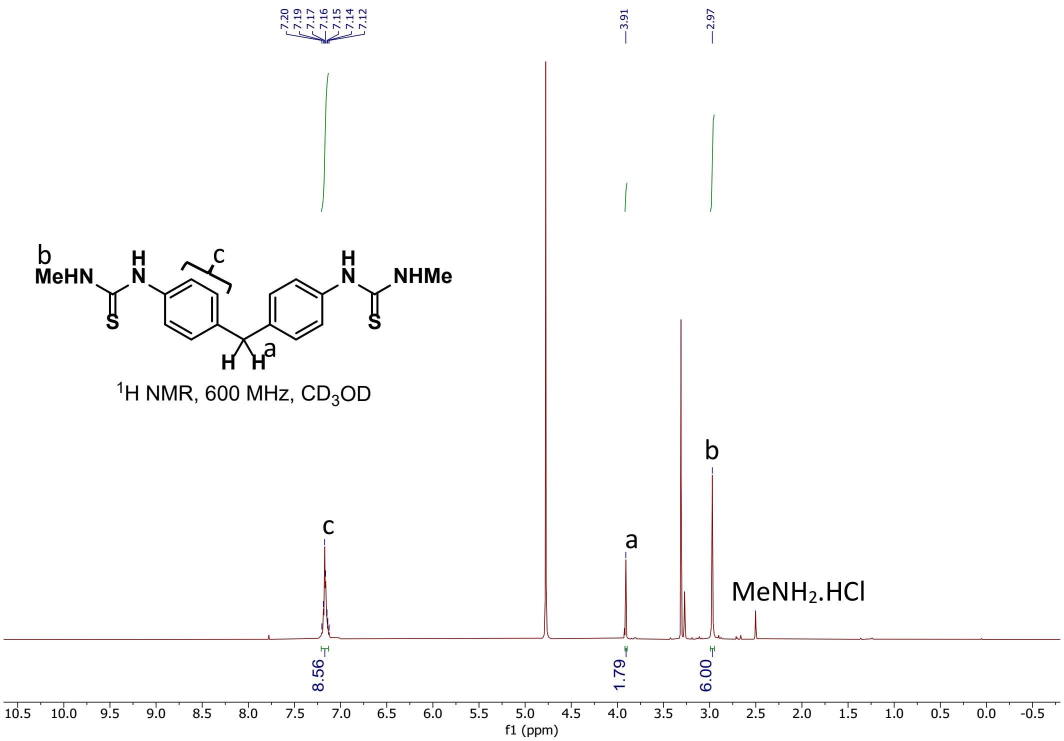

Figure S24. ^{1}H NMR spectrum of compound **1d**

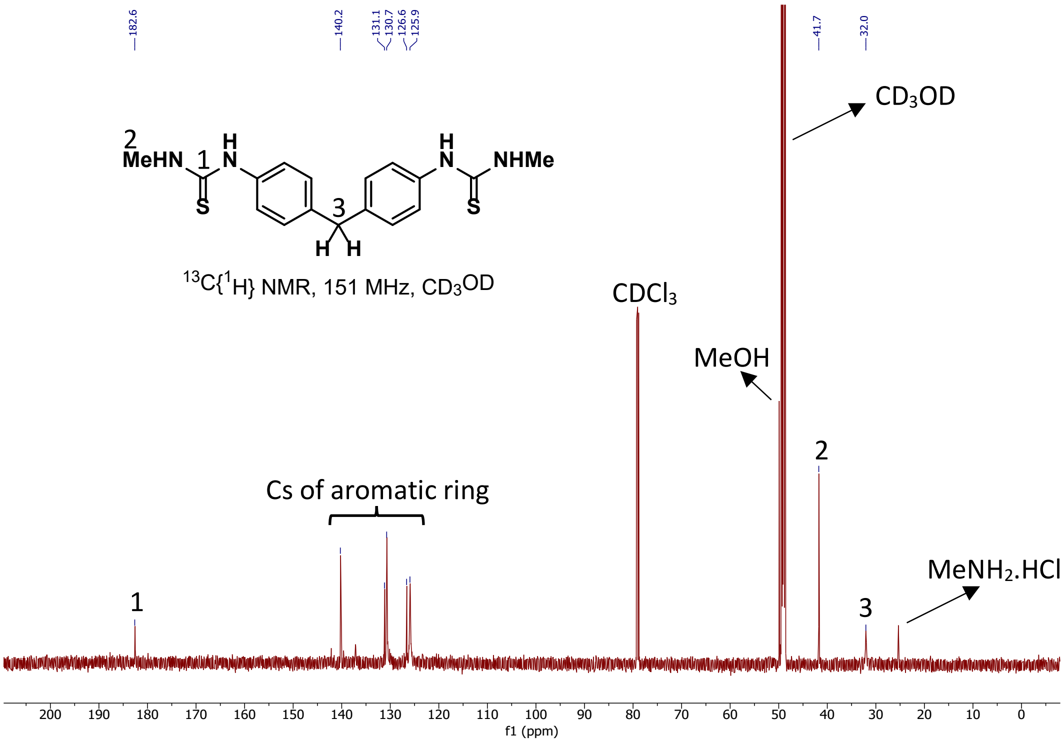

Figure S25. ^{13}C NMR spectrum of compound **1d**

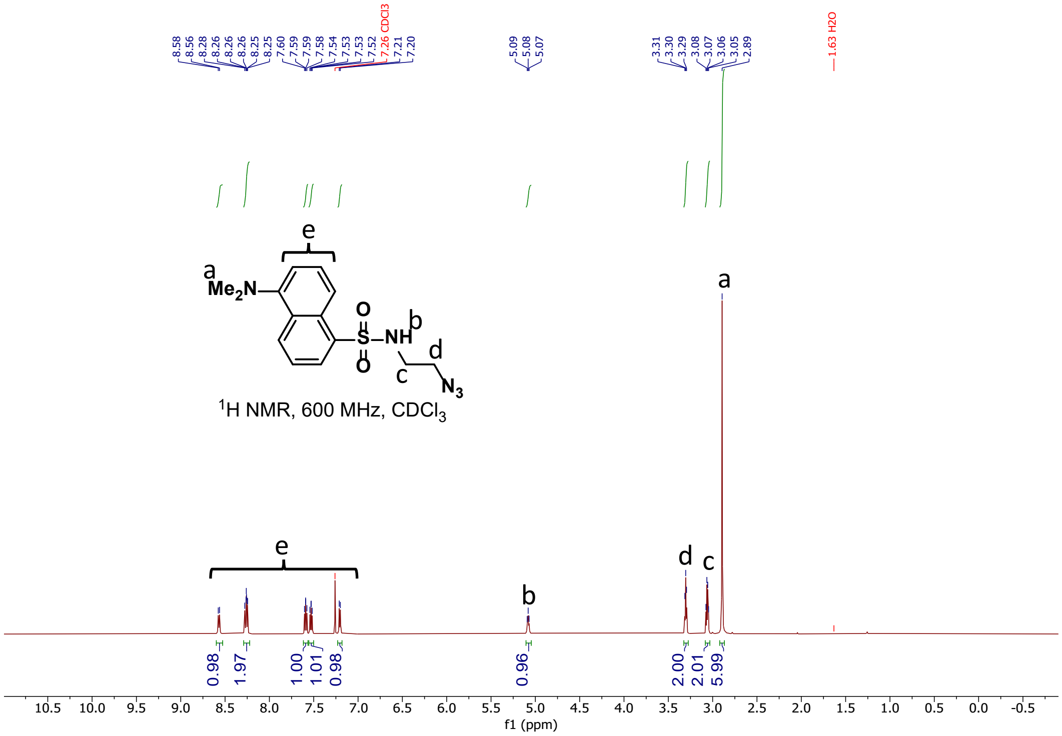

Figure S26. ^{1}H NMR spectrum of compound **S4**

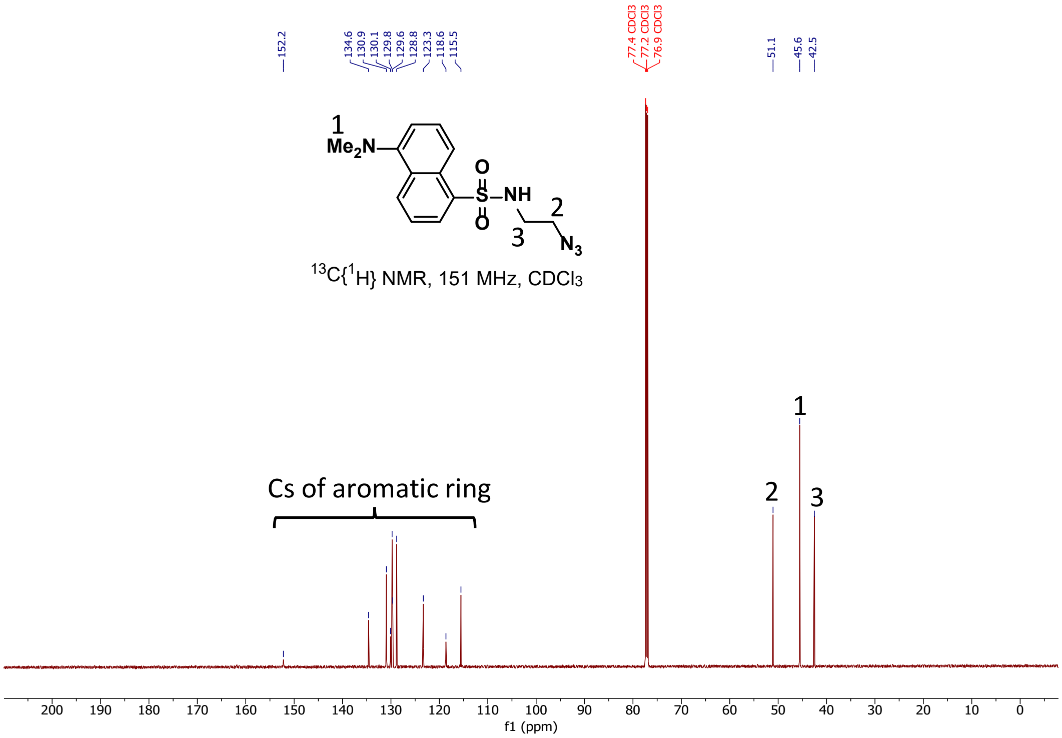

Figure S27. ^{13}C NMR spectrum of compound **S4**

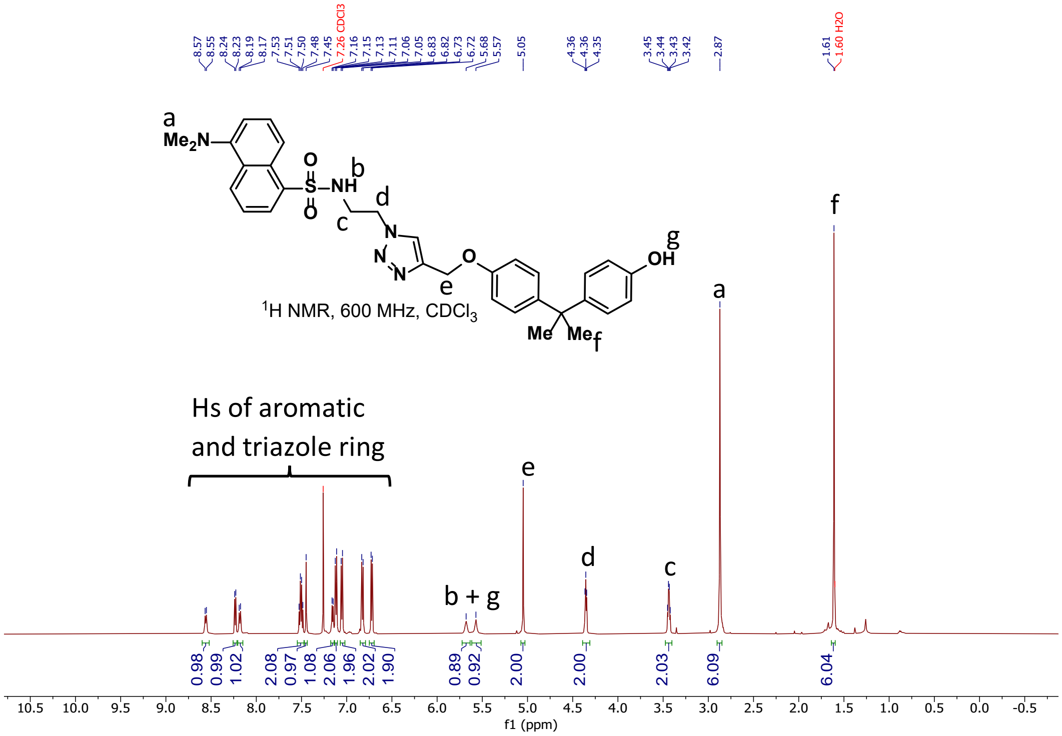

Figure S28. ^{1}H NMR spectrum of compound **S6**

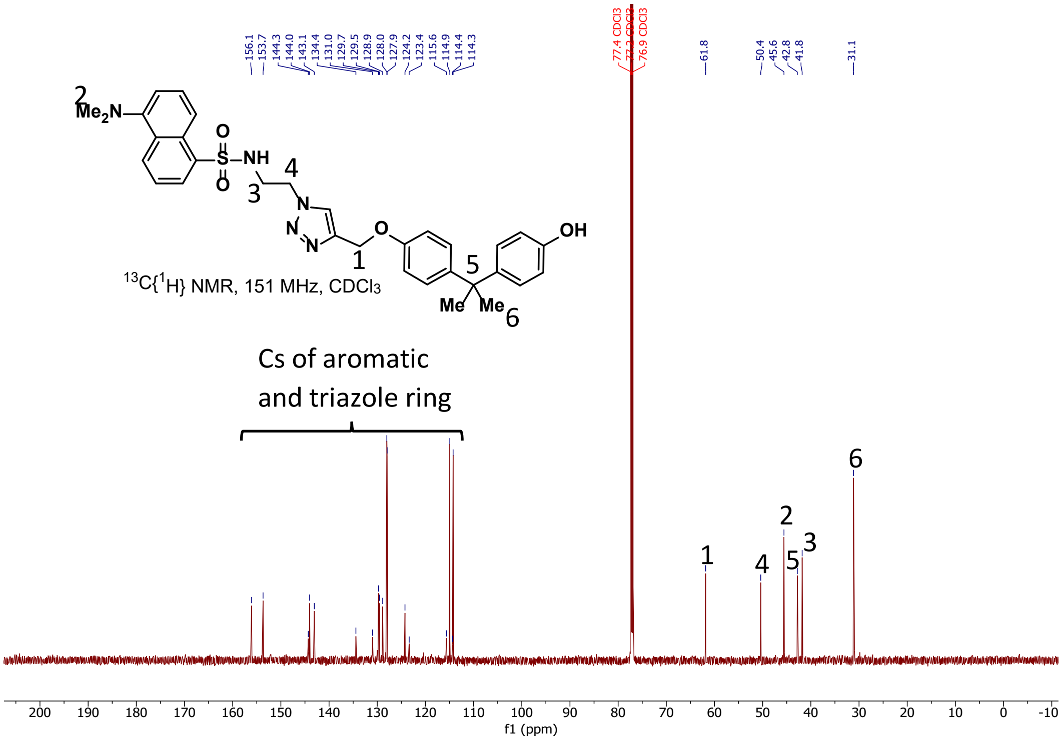

Figure S29. ^{13}C NMR spectrum of compound **S6**

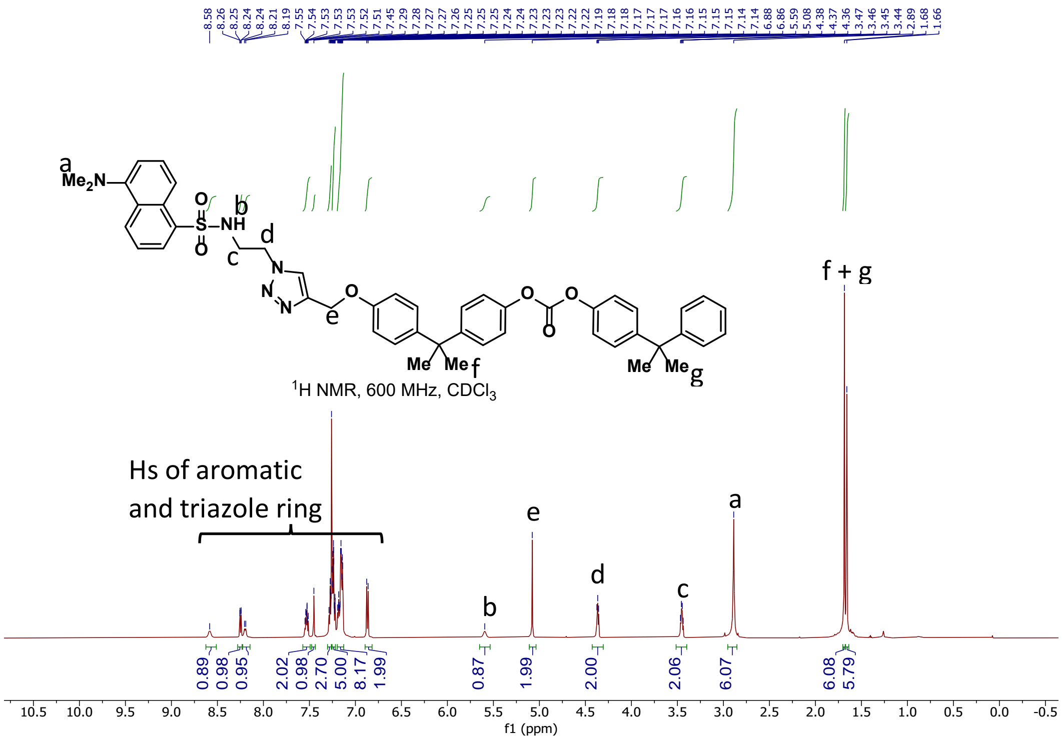

Figure S30. ^{1}H NMR spectrum of compound **5**

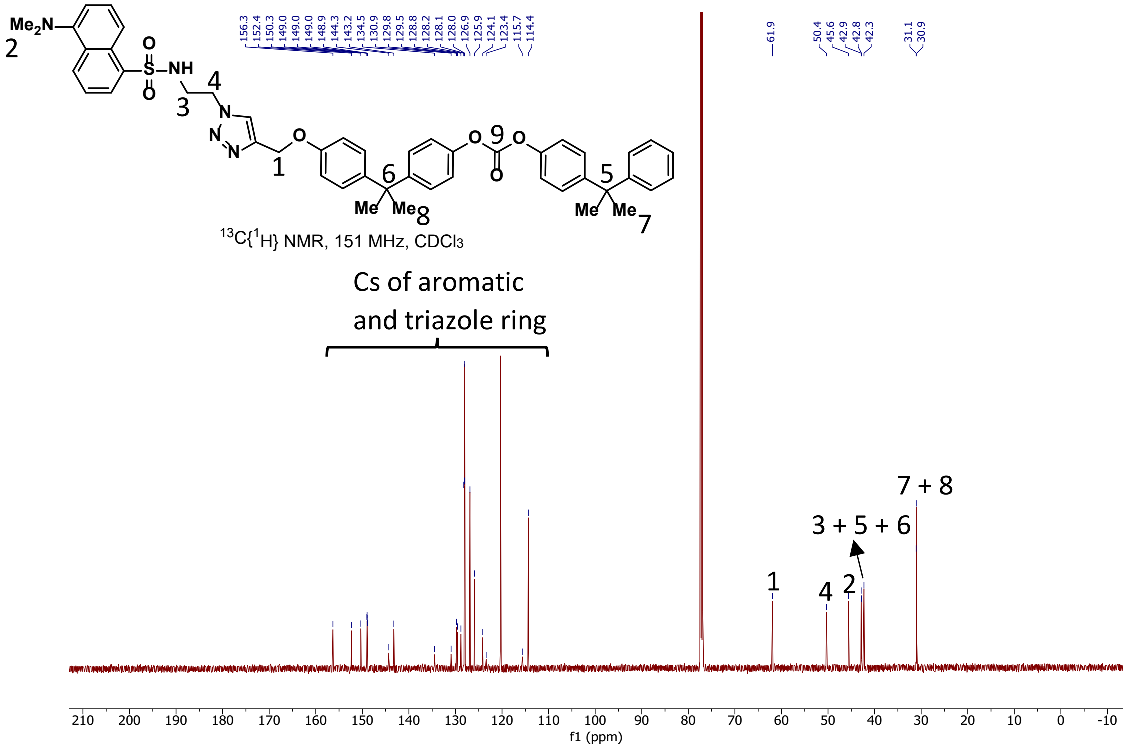

Figure S31. ^{13}C NMR spectrum of compound **5**

Mass spectra

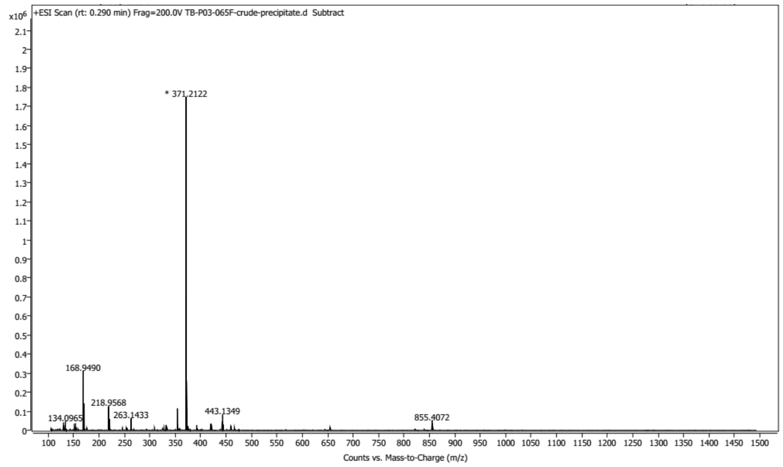

Figure S32. HRMS-ESI spectrum of compound **1a**

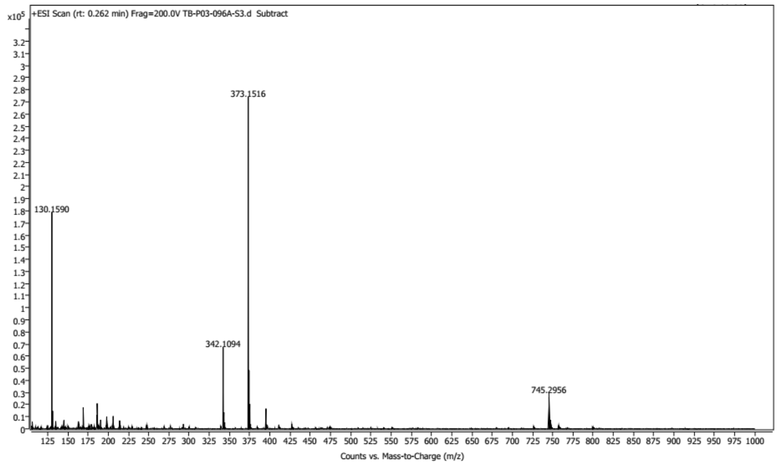

Figure S33. HRMS-ESI spectrum of compound **1b**

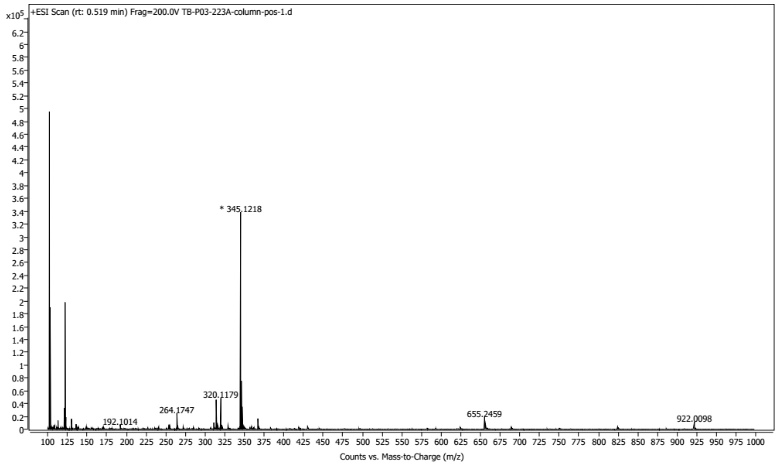

Figure S34. HRMS-ESI spectrum of compound **1d**

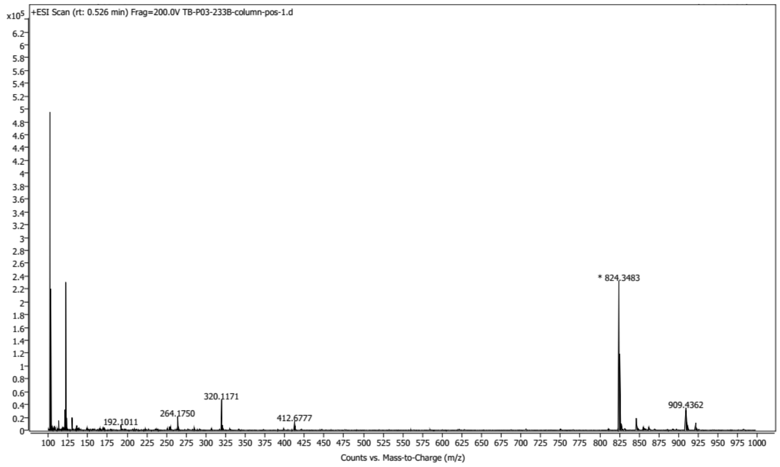

Figure S35. HRMS-ESI spectrum of compound **5**

References

(1) Awino, J. K.; Zhao, Y. Protein-Mimetic, Molecularly Imprinted Nanoparticles for Selective Binding of Bile Salt Derivatives in Water. *Journal of the American Chemical Society* **2013**, *135*, 12552-12555.

(2) Seuring, J.; Reiss, P.; Koert, U.; Agarwal, S. Synthesis, characterization and properties of a new polymerisable surfactant: 12-Methacryloyl dodecylphosphocholine. *Chemistry and Physics of Lipids*, **2010**, *163*, 367-372.

(3) Zhao, Y.; Zhong, Z. Oligomeric Cholates: Amphiphilic Foldamers with Nanometer-Sized Hydrophilic Cavities. *Journal of the American Chemical Society,* **2005**, *127*, 17894-17901.

(4) Xiao, Z.; Song, S.; Chen, D.; van Merkerk, R.; van Der Wouden, P. E.; Cool, R. H.; Quax, W. J.; Poelarends, G. J.; Melgert, B. N.; Dekker, F. J. Proteolysis targeting chimera (PROTAC) for macrophage migration inhibitory factor (MIF) has anti-proliferative activity in lung cancer cells. *Angewandte Chemie International Edition* **2021**, *60*, 17514-17521.

(5) Ryu, E. H.; Zhao, Y. 2005. Efficient synthesis of water-soluble calixarenes using click chemistry. *Organic Letters*, **2005**, *7*, 1035-1037.

(6) Lewandowski, B.; De Bo, G.; Ward, J. W.; Papmeyer, M.; Kuschel, S.; Aldegunde, M. J.; Gramlich, P. M.; Heckmann, D.; Goldup, S. M.; D'Souza, D. M.; Fernandes, A. E. 2013. Sequence-specific peptide synthesis by an artificial small-molecule machine. *Science*, **2013**, *339*, 189-193.

(7) Schaufelberger, F.; Seigel, K.; Ramström, O. Hydrogen-Bond Catalysis of Imine Exchange in Dynamic Covalent Systems. *Chemistry–A European Journal*, **2020**, *26*, 15581-15588.

(8) Stacy, D. M.; Le Quement, S. T.; Hansen, C. L.; Clausen, J. W.; Tolker-Nielsen, T.; Brummond, J. W.; Givskov, M.; Nielsen, T. E.; Blackwell, H. E. Synthesis and biological evaluation of triazole-containing *N*-

acyl homoserine lactones as quorum sensing modulators. *Organic & Biomolecular Chemistry*, **2013**, *11*, 938-954.

(9) Ye, Y. S.; Huang, Y. J.; Chang, F. C.; Xue, Z. G.; Xie, X. L. Synthesis and characterization of thermally cured polytriazole polymers incorporating main or side chain benzoxazine crosslinking moieties. *Polymer Chemistry*, **2014**, *5*, 2863–2871.

(10) Silver, R.; Nirpal, A. K.; Sathyamoorthi, S. Taming tethered nitreniums for alkene functionalization reactions. *Journal of Organic Chemistry*, **2024**, *89*, 15352–15357.

(11) Saura, A. V.; Marin, M. J.; Burguete, M. I.; Russell, D. A.; Galindo, F.; Luis, S. V. The synthesis of new fluorescent bichromophoric compounds as ratiometric pH probes for intracellular measurements. *Organic & Biomolecular Chemistry*, **2015**, *13*, 7736–7749.

(12) Bahrami, F.; Zhao, Y. Rational design and synthesis of an artificial enzyme for S_N2 reactions through micellar imprinting. *Organic Letters*, **2024**, *26*, 73–77.

(13) Batey, R. A.; Santhakumar, V.; Yoshina-Ishii, C.; Taylor, S. D. An efficient new protocol for the formation of unsymmetrical tri- and tetrasubstituted ureas. *Tetrahedron Letters*, **1998**, *39*, 6267–6270.
